# Effective Dementia Education and Training for the Health and Social Care Workforce: A Systematic Review of the Literature

**DOI:** 10.3102/0034654317723305

**Published:** 2017-07-31

**Authors:** Claire A. Surr, Cara Gates, Donna Irving, Jan Oyebode, Sarah Jane Smith, Sahdia Parveen, Michelle Drury, Alison Dennison

**Affiliations:** Leeds Beckett University; University of Bradford

**Keywords:** Alzheimer’s, staff training, workforce development, critical interpretive synthesis

## Abstract

Ensuring an informed and effective dementia workforce is of international concern; however, there remains limited understanding of how this can be achieved. This review aimed to identify features of effective dementia educational programs. Critical interpretive synthesis underpinned by Kirkpatrick’s return on investment model was applied. One hundred and fifty-two papers of variable quality were included. Common features of more efficacious educational programs included the need for educational programs to be relevant to participants’ role and experience, involve active face-to-face participation, underpin practice-based learning with theory, be delivered by an experienced facilitator, have a total duration of at least 8 hours with individual sessions of 90 minutes or more, support application of learning in practice, and provide a structured tool or guideline to guide care practice. Further robust research is required to develop the evidence base; however, the findings of this review have relevance for all working in workforce education.

The care of people with dementia is of global concern (Alzheimer’s Disease International, 2010; World Health Organization and Alzheimer’s Disease International, 2012). People with dementia account for two thirds of U.K. care home residents (Knapp et al., 2007) and occupy around one quarter of acute hospital beds (Alzheimer’s Society, 2009) and have more hospital and skilled nursing facility stays and home health care visits in the United States than older people generally (Alzheimer’s Association, 2015). However, care quality concerns have been raised, including the adequacy of workforce skills and knowledge to provide effective care (Department of Health, 2009; U.S. Department of Health and Human Services, 2013). In the United Kingdom, policy initiatives (Department of Health, 2009, 2012) have aimed to address this skills gap, leading to increases in dementia education and training provision. However, there remains limited available evidence of education and training efficacy. Concern with the effective transfer of knowledge developed within education settings, to the workplace and in particular the realistic connection of theory to practice, has been a growing concern within education research (Smith, 2003). However, to date limited attention has been given to the specific professional development needs of the dementia workforce and effective approaches to ensuring this skills gap is met. Similar initiatives to increase specialist skills or clinical expertise into the broader workforce occur frequently in health care, for example, mental health, end-of-life care (Department of Health, 2016). Therefore, a review of the existing evidence base, to inform future dementia workforce development initiatives, is required. Similar issues with workforce skills gaps are found in many sectors including manufacturing, technology and services, food and agriculture (Creaser, Hargreaves, & Ong, 2014; Dychtwald, Erikson, & Morison, 2006) internationally, and thus the results are likely to have applicability to broader, professional development and adult, lifelong and workplace education.

## Background

There has been increasing emphasis placed on ongoing knowledge and skills development of workforces, with the emergence of knowledge-based economies (Dall’Alba & Sandberg, 2006). The health and social care workforce is diverse in its previous access to and experience of postsecondary education. The majority of the dementia care workforce is unqualified, low paid, low status and has no clear career path. In the United Kingdom and internationally within social care particularly, there are low levels of literacy and numeracy and many staff have English as a second, or additional, language (All-Party Parliamentary Group on Dementia, 2009; Wilson, 2014), in part due to an increasing reliance on migrant workers (Bettio, Simonazzi, & Villa, 2006; Bourgeault, Atanackovic, Rashid, & Parpia, 2010; Cangiano & Shutes, 2010). Conversely, health professions are predominantly degree-qualified roles, but specialisms may provide limited provision of, or access to, dementia-specific education. This diversity within the workforce of existing dementia knowledge and previous educational exposure has required employers to engage with provision of workplace learning opportunities. While further and higher education institutions have met some of this demand, a significant amount of dementia-training provision is provided in-house or by private providers.

In England, currently there is no requirement for continuing professional development education or training to be accredited, and a nationally agreed sector framework for dementia educational content and learning outcomes was only published in October 2015 (Health Education England Skills for Health and Skills for Care, 2015). A similar picture is apparent internationally with recent publication of competency or education and training frameworks (Care Council for Wales, 2016; Health and Social Care Board, 2016; Traynor, Cumming, & Britten, 2015), but little further support or provision for regulation or quality monitoring. As a result, the content and quality of dementia training and education in England is variable and low levels of dementia knowledge remain commonplace (Royal College of Psychiatrists, 2011). The government targets for numbers of NHS staff trained on dementia (Department of Health, 2013, 2014) may, in some cases, have led to a volume rather than quality or efficacy-driven approach. Therefore, greater understanding and consideration of what effective dementia education and training for this workforce entails, is required. This is particularly important if this imperative to educate the workforce is to lead to improved outcomes for people with dementia. The challenges experienced with dementia care workforce development mirror those found in development of the workforce across other professional sectors, including teaching, business, and the social and natural sciences (Webster-Wright, 2009). While there are differences between the roles of those providing health and social care to people with dementia and other professions, for example teaching, there are also many parallels of relevance to the continuing professional development of these professional groups including, the diversity of education of the workforce (i.e., qualified teachers/health professional vs. unqualified staff/assistants), working within an organizational context and culture governed by external guidelines and quality standards and assurance, team-based working, and the need to translate theoretical and knowledge-based learning into real-world practice. In the practice setting, both may be under pressure to adopt a task-focused approach in order to achieve targets in a time and resource pressured environment and in what is an unpredictable setting, with complex group dynamics. Opfer and Pedder (2011) have argued that teacher education must be conceptualized as a complex system, with various dynamics at the individual (teacher), meso (institutional), and macro (school system) levels at play in influencing under what conditions teachers learn, why, and how. Thus, we argue that health and social care workforce education too, should be conceptualized as a complex system, with many facets at the individual, meso, and macro levels at play that must be understood in understanding learning processes.

The effective transfer of theoretical education to practice settings has received much attention in educational practice and research (Allen & Wright, 2014; Kessels & Korthagen, 1996) and has been a particular concern within the initial training and ongoing professional development of the health and social care workforce (Corlett, 2000; Jackson, Bluteau, & Furlong, 2013; Landers, 2000; Scully, 2011; Upton, 1999). In particular, effective methods to bridge the gap between the desired practices taught in the classroom and the reality of working in real-life health and social care practice have challenged those delivering health education (Jackson et al., 2013). Attempts to address the perceived gap within health and wider education research have included a focus on the design and content of educational programs, the knowledge and skills of the educator/teacher, the teaching and learning processes utilized (i.e., delivery methods and assessment strategies; Gallagher, 2004), the use of critical reasoning skills and reflection by learners who must be supported to be proactive in building bridges between theory and practice (Hatlevik, 2011; Severinsson, 1998; Wilkinson, Smallidge, Boyd, & Giblin, 2015) and use of simulation (Roth, Mavin, & Dekker, 2014; Wall, Andrus, & Morrison, 2014). However, little is known about whether such methods also apply to dementia education and practice in the context of workplace learning.

Workplace learning has been conceptualized in a variety of ways, which recognize the need to acquire appropriate attitudes, conceptual knowledge, and practical skills that lead to the development of practical competencies (Dall’Alba & Sandberg, 2006), and research suggests that specific forms of learning may be preferred by learners located in the workplace (Smith, 2003), as opposed to other types of education. In particular, Smith argues learners have a reducing requirement for a proximal guide/facilitator and a greater need for interaction and construction as their expertise grows. Dreyfus’s (1982) model of five stages to skill development (Stage 1: novice to Stage 5: expert), applied to nurse education by Benner (1982), highlights the role of both knowledge and experience in guiding appropriate practice and decision making. It connects theoretical knowledge with practical experience in task performance, with novice practitioners being characterized by being unable to deviate from taught rules to guide action and experts being those who are able to work holistically drawing on both formal educational preparation and acquired experience to inform practice. Thus, expertise is context specific and more advanced skills can only be achieved through experience in real-world situations (Dall’Alba & Sandberg, 2006).

The diversity of the dementia care workforce means those requiring workplace education may be at varying stages of proficiency with regard to exposure to both clinical work with people with dementia and dementia education. Some learners may, for example, have a degree or postgraduate qualification in a specialist area of clinical practice (e.g., nursing, physiotherapy, radiography, medicine) and many or few years’ clinical experience, but limited exposure to specialist care of people with dementia. Other learners may work daily in specialist dementia care but have had limited previous exposure to formal clinical or practice education (e.g., social care workers, nursing assistants/auxiliaries). This has implications for educational approaches adopted, since learners’ preferred teaching and learning methods and those required for effective learning may differ depending on degree of learner expertise (Smith, 2003). This can be particularly challenging for educators who may need to provide professional development to groups of learners who span a range of levels of expertise, whether this is in health and social care education or professional development and workplace learning in another profession. Therefore, an understanding of teaching and learning methods that motivate learners and which support attitude and practice change is required.

Despite the body of educational theory and research on professional and workplace education, understanding of what constitutes effective education and training for the dementia workforce is poorly understood and seldom considered when developing programs. To date, a number of systematic reviews have examined the evidence base underpinning dementia education and training interventions for the health and social care workforce. However, they are limited by their focus on only one aspect of efficacy (K. E. J. Elliott, Scott, Stirling, Martin, & Robinson, 2012); a single pedagogical approach (Brody & Galvin, 2013; Fossey et al., 2014); efficacy within a single workforce group or setting (Alushi, Hammond, & Wood, 2015; Beeber, Zimmerman, Fletcher, Mitchell, & Gould, 2010; Kuske et al., 2007; Perry et al., 2011; Tullo & Allan, 2011); or a single aspect of care (Eggenberger, Heimerl, & Bennett, 2013; McCabe, Davison, & George, 2007; Raymond et al., 2014; Spector, Orrell, & Goyder, 2013; Zientz et al., 2007). Overall existing reviews consistently note the generally poor quality of existing research and variability in results, with some reported benefits of education and training particularly in areas such as staff knowledge and attitudes but no consistency across studies.

Kirkpatrick’s (1979, 1984) “Return on Investment” model developed specifically for evaluation of training consists of four levels and is widely adopted in the evaluation of training and education provision (Bates, 2004).

- Level 1: Learner’s *reaction* to and satisfaction with the program
- Level 2: The extent to which *learning* has occurred including knowledge, skills, confidence, and attitude change
- Level 3: The extent to which staff *behavior* or practices have changed as a result of completing the program
- Level 4: The *results* or outcomes that have occurred because of training

Criticisms of Kirkpatrick’s model have been presented including the following: being oversimplified and incomplete in its understanding of the transfer of learning to practice; implying a hierarchy of evidence with behavioral and outcome change deemed more important to measure than reaction; assuming that each level of the model is associated with the previous and following level, without evidence to support this; and a lack of empirical testing (Giangreco, Carugati, & Sebastiano, 2008; Holton, 1996; Tamkin, Yarnall, & Kerrin, 2002). While Kirkpatrick’s model has been subject to such critique, it remains a widely applied approach to considering the levels at which it is helpful to evaluate training, in order to understand potential return on investment.

Brody and Galvin (2013) highlight that much of the published dementia education research fails to evaluate impact across more than one level within Kirkpatrick’s framework, meaning any relationship between levels, for example, between changes to staff knowledge and outcomes for people with dementia, is not understood. All of the systematic reviews on dementia education and training conclude further research is required. While some reviews identify features present in efficacious training programs, none examines these across each of the four Kirkpatrick levels of evaluation or combine them to provide an overall, wider picture of the ingredients most likely to lead to efficacy.

## Aims

The aims of this review were to identify the factors associated with effective dementia education and training for health and social care staff, across service settings in order to draw out the implications for those involved in dementia education and more broadly for all with an interest in workforce education and training.

## Method

### Protocol

The review protocol is registered with the PROSPERO international prospective register of systematic reviews (CRD42015027475).

### Search Strategy

Search strategies were agreed by the authorship team based on keywords developed from initial scoping searches. The following databases were searched: MEDLINE, PsycINFO, CINAHL, AMED, British Education Index, Education Abstracts, ERIC (EbscoHost), The Cochrane Library–Cochrane reviews, Economic evaluations, CENTRAL (Wiley), HMIC (Ovid), ASSIA, IBSS (Proquest), and Conference Proceedings Citation Indexes (Web of Science). Searches consisted of a combination of text words and subject headings for the following themes: Dementia/Alzheimer’s, training/education, staff knowledge, and patient outcomes. Reference lists of key papers and e-alerts were used to include additional articles published between search completion and the end of November 2015.

### Procedure

**Figure 1.**
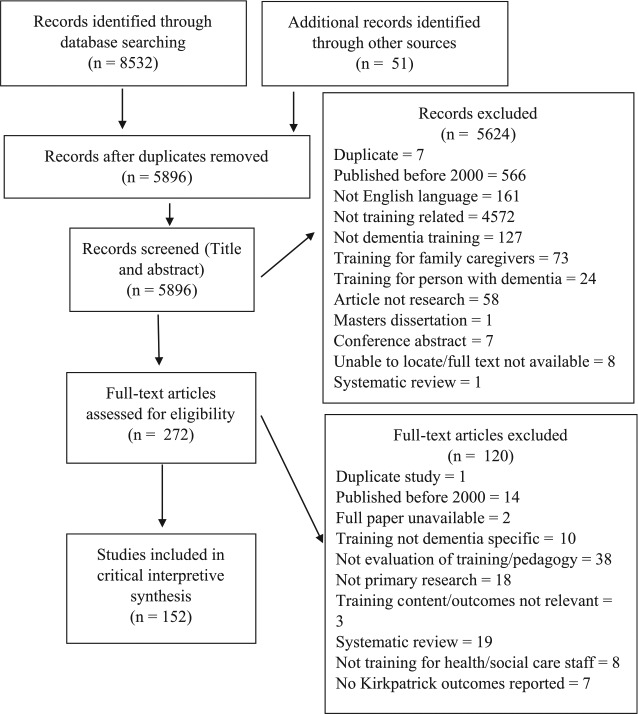
PRISMA (Preferred Reporting Items for Systematic Reviews and Meta-Analyses) diagram of included and excluded studies.

All hits were downloaded into Endnote software, where duplicate entries were removed. Papers were excluded based on an initial title screen for relevance (see Figure 1), followed by abstract review of potentially relevant papers and finally full paper review of remaining articles. Inclusion criteria were studies written in English and published between 2000 and April 2015, whose focus was research evaluating a dementia education or training program. Exclusion criteria were conference abstracts, masters’ dissertations, and training programs for family caregivers or people with dementia. Data extraction from all relevant papers was completed using a standard template in Excel format (see Supplemental Tables S1 and S2, available in the online version of the journal, for data extraction headings).

At the full-text review/data extraction stage, to ensure included papers were relevant to answering the review aims, further inclusion criteria were added. They were the following: study reports on primary research, evaluates a dementia training program or pedagogical approach to delivery of the training, is delivered to staff working in health or social care settings and reports on at least one of Kirkpatrick’s (1984) four levels of training evaluation.

### Analysis

Data analysis was conducted using critical interpretive synthesis (CIS; Dixon-Woods et al., 2006), a nontraditional systematic review method that draws on systematic qualitative enquiry, incorporating interpretive approaches. CIS permits synthesis of large amounts of diverse literature. CIS is particularly useful when studies to be reviewed use different research methods, stem from a range of disciplines and where the review is intended to inform generation of theory, evidence-based practice, and decision making. CIS involves a process of synthesis of the evidence, involving understanding studies in relation to themselves and other studies through thematic and conceptual comparison (Kangasniemi, Kallio, & Pietila, 2014). The product of this synthesis is not aggregation of data, as in a traditional systematic review, but the generation of new knowledge in the form of overarching themes, grounded in the studies included in the review (Dixon-Woods et al., 2006). Another feature of the review process is the selection of papers that are most relevant to explaining or theory building around the concept(s) within the review, rather than on selection of papers utilizing a specific methodology or methods. Therefore, exclusion of studies on the basis of quality is not undertaken, except in the case of those deemed to be “fatally flawed” (Dixon-Woods et al., 2006). Given the expected diverse nature of the evidence base surrounding effective dementia education and training and the review aims to develop new knowledge in the form of overarching themes, regarding the most effective approaches to adopt, CIS was chosen as the most appropriate review method. The analytic process and knowledge building was underpinned by Kirkpatrick’s (1984) four-level model for the evaluation of training interventions.

### Quality Review

All included papers were subject to a quality review using an adapted version of criteria developed by Caldwell, Henshaw, and Taylor (2005) and the Critical Skills Appraisal Programme (2014) with a maximum possible quality score of 14 (see Table 1). In line with CIS, this was not used to exclude papers but to provide a description of quality of the evidence base, as part of the analytic process. Papers were provided with an overall quality rating of high (score 11–14), medium (score 6–10), or low (score ≤5). Papers with an overall rating of “high” necessarily met all or the majority of the reporting/quality criteria to at least an adequate level and thus a degree of trust can be attributed to their findings. Papers with a rating of “low” met few of the reporting or research quality criteria and therefore, their results, while potentially interesting or relevant require treating with some caution. An initial sample of 15 papers was rated independently for quality, using the adapted criteria, by two of the authors CS and CG. The ratings were compared, disagreements discussed, and an agreed rating decided for each paper. Following this, a further five studies were independently rated by both authors and compared to assess for interrater agreement, which was achieved to a satisfactory level (1 point or less difference) on all papers. Following this CS and CG each undertook quality ratings for half of the remaining papers.

**Table 1:** Adapted quality rating criteria

*Quality criteria* *are* . . .	*Specific questions to consider when rating*	*Rating*
1. Are the research aims and questions/hypotheses clearly stated?	• Do(es) the author(s) clearly state what they plan to research?	0 = no; 1 = partially; 2 = yes
2. Are ethical issues addressed?	• Do(es) the author(s) state that ethical approval was sought?	0 = no; 1 = partially; 2 = yes
	• Do(es) the author(s) demonstrate an awareness of the ethical issues raised by the study? (e.g., informed consent, confidentiality, responding to upset or distress, withdrawal etc.).	
3. Are the methodology/study design appropriate to the research question and rationale for choice evident?	• Does the author explicitly state what research methodology they have chosen?	0 = no; 1 = partially but with weaknesses/missing info; 2 = yes
	• Is the chosen methodology appropriate to the research question? For example, qualitative or quantitative or mixed-methods approach? Where qualitative—grounded theory, interpretative phenomenological analysis, ethnography etc.	
	• For qualitative research, does the chosen methodology appear appropriate to the research aims and questions? Is this fully justified?	
	• For quantitative research, do(es) the author(s) clearly state the design of the study? Do(es) the author(s) justify the research design used? (e.g., longitudinal, cross-sectional, etc.) Do(es) the author(s) identify the main variables investigated in the study?	
4. Are the sample size, selection and description appropriate?	• Do(es)the authors(s) clearly state how the study sample size was identified?	0 = no; 1 = partially but with weaknesses/missing info; 2 = yes
	• Do(es)the sample size appear to be large enough/appropriate?	
	• Do(es) the author(s) adequately describe the sample (e.g., gender, age, relationship to care receiver, etc.) so that the reader can determine transferability of findings?	
	• Do(es) the author(s) describe the context of where the samples were recruited from?	
	• Do(es) the author(s) describe the method of recruitment used? (e.g., the sampling method, recruitment, etc.)	
	• Do(es) the author(s) identify the inclusion criteria?	
5. Are the method(s) of data collection appropriate, reliable, and valid?	• For quantitative studies	0 = no; 1 = partially but with weaknesses/missing info; 2 = yes
	○ Do(es) the author(s) justify that the measure is suitable for this population?	
	○ Do(es) the author(s) use measures that measure the desired constructs?	
	○ Do(es) the author(s) indicate whether the measures used have good psychometric properties? (e.g., test–retest reliability, interrater reliability, internal reliability, and internal consistency [Cronbach’s alpha]).	
	○ Do(es) the author(s) indicate that the measures used have demonstrated validity?	
	• For qualitative studies	
	○ Were the methods used appropriate for the participants, valid, and likely to be free of bias?	
	○ Do(es) the author justify why particular data collection approaches were used, for example, interviews, focus groups?	
6. Are the method(s) of data analysis reliable and valid?	• For quantitative studies	0 = no; 1 = partially; 2 = yes
	○ Do(es) the author(s) state which statistic tests were used?	
	○ Do(es) the author(s) use statistical tests that appear to be appropriate to the nature of the data collected? (e.g., Do the data meet the assumptions of the test?)	
	○ Were the statistical tests used appropriate to the research question?	
	○ Do(es) the author(s) consider the impact of extraneous variables and control for these within the analysis process?	
	○ Do(es) the author(s) provide evidence of statistical findings? (e.g., data within the text, tables, etc.).	
	○ Do(es) the author(s) state the levels of significance?	
	• For qualitative studies	
	○ Do(es) the authors(s) state what approach they used to data analysis?	
	○ Does this approach appear to be suitable to the data gathered?	
	○ Does the approach appear to have been implemented in a structured/robust manner?	
	○ Do(es) the author(s) provide details of how findings were validated?	
7. The findings and discussion clearly stated and appropriate?	• Does the author(s) explicitly state their findings?	0 = no; 1 = partially; 2 = yes
	• Do(es) the author(s) present the statistical/qualitative data in a clear manner?	
	• For quantitative studies	
	○ Do(es) the author(s) clearly differentiate between significant and nonsignificant findings?	
	• For qualitative studies	
	○ Do(es) the author(s) clearly identify key themes or issues arising from the data?	
	○ Do(es) the author(s) present data to support the themes presented?	
	• Do(es) the author(s) summarize the main findings?	
	• Do(es) the author(s) link their findings back to the research aims?	
	• Do(es) the author(s) link their findings current literature and/or psychological theory?	
	• Do(es) the author(s) consider the clinical usefulness of their findings?	
	• Do(es) the author(s) identify the limitations of the research? (e.g., sample size, recruitment strategies, method of data collection, analysis, etc.)	
	• Do(es) the author(s) identify the strengths of the research? (e.g., its usefulness, etc.)	
	• Do(es) the author(s) make conclusions that are supported by their discussions of their findings?	
Total		Range: 0–14

## Results

In total 152 papers were included in the review (see Figure 1). A table of included studies can be found in Supplementary Table S1 and further details on training approaches, quality and Kirkpatrick outcomes are reported in Supplementary Table S2 (available in the online version of the journal).

### Summary of Studies

#### Setting and Participants

Table 2 provides an overview of the 152 included papers. The majority originated from the United States (38%) and the United Kingdom (20%). Training programs were predominantly delivered to staff working in care homes (49%) and were aimed at nurses (34%) and nursing assistants/aides (37%).

**Table 2:** Characteristics of included studies

Description	*N* (%)
Country of study	
USA	58 (38)
UK	30 (20)
Australia	17 (11)
Canada	18 (12)
Netherlands	5 (3)
Norway	5 (3)
Sweden	4 (3)
France	3 (2)
Rest of Europe	8 (5)
Other	4 (3)
Setting	
Care homes	75 (49)
Hospitals	14 (9)
Higher education	18 (12)
Primary care	12 (8)
Community	7 (5)
Assisted living	5 (3)
Day care	2 (1)
More than one service setting	19 (13)
Staff group training aimed at	
Nurse aides/assistants	56 (37)
Unknown staff groups within a particular service setting	53 (35)
Qualified Nurses	51 (34)
General practitioners/doctors/physicians	23 (9)
University students on health/social care programs	22 (14)
Managers	15 (10)
Activities staff	13 (9)
Social workers	13 (9)
Ancillary staff (porters, laundry, etc.)	11 (7)
Allied health professionals	9 (6)
Pharmacists	3 (2)
Unknown	2 (1)
Methodology	
Quantitative	97 (64)
Qualitative	21 (14)
Mixed methods	34 (22)
Quality	
High	52 (34)
Medium	79 (52)
Low	21 (14)

#### Methodology and Quality

The majority of studies adopted a quantitative methodology (63%). One third (34%) were rated as high quality, just over half (52%) medium quality, and 14% were rated as low quality. Across all three study designs (qualitative, quantitative, and mixed methods), the largest proportion of studies were rated as moderate quality. Despite parity of quality criteria across all study designs, quantitative studies had the largest proportion rated as high quality (38% of all quantitative studies) and the lowest rated as low quality (11%). Qualitative studies had the lowest overall proportion rated as high quality (23%) and the highest rated as low quality (32%).

**Figure 2.**
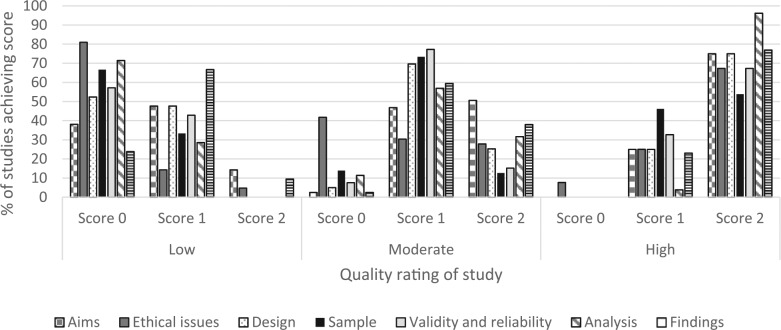
Scores on quality review tool individual criteria by overall quality of study.

A breakdown of the individual quality criteria scores by overall quality rating of studies (low, moderate, and high) is provided in Figure 2. Low-quality studies were particularly poor at providing details of ethics issues and considerations, with over 80% of studies scoring 0 on this criterion; using appropriate data analysis techniques or reporting on the analysis techniques used (70%+ of studies scoring zero); and recruiting an appropriate sample size, or providing adequate detail of the sample (65%+ scoring 0). However, over 40% of moderate-quality studies scored zero on adequate reporting of ethical issues and less than 15% of studies scored a two on appropriate sample size and reporting of sample characteristics, and reliability and validity of data collection methods used. Moderate-quality studies were stronger on clarity of reporting of study aims and objectives, with over half of studies scoring two on this criterion. High-quality studies were characterized by each criteria being awarded a score of two for 50% or more of studies. However, over 40% of high-quality studies, scored one for an appropriate sample size and reporting of sample characteristics, and 8% scored a zero for adequate reporting of ethical issues.

#### Teaching and Learning Methods Adopted

Small or large group face-to-face delivery was the most common approach to training/educational delivery, adopted in 120 (79%) studies (Table 3). This approach included a diverse range of teaching and learning methods including didactic lectures, discussion, video clips, exercises, and activities. In 45% of studies, classroom teaching alone was used and in 34% of studies, this was used alongside other teaching methods.

**Table 3:** Teaching and learning approaches adopted

Teaching and learning approach	*N* (%)^a^
Small/large group face-to-face	69 (45)
Small/large group face-to-face plus	51 (34)
In-service/practice-based learning	25 (16)
Mentorship/supervision	6 (4)
Experiential learning/simulation	7 (5)
Practical exercise/project	5 (3)
Written materials	3 (2)
Psychoeducation	1 (1)
Online learning	1 (1)
DVD	1 (1)
Drama	1 (1)
Peer support	1 (1)
Individual/group DVD	9 (6)
Written resource	6 (4)
Online	5 (3)
In-service	4 (3)
Train-the-trainer	3 (2)
Peer support/learning set	2 (1)
Simulation/experiential	2 (1)
Drama	2 (1)
Counselling	2 (1)
Psychoeducation	1 (1)

a Number of studies is greater than 152 since some studies compared more than one type of training.

### Staff Reactions to Training

Seventy-four of the 152 papers (49%) reported on learners’ reactions to the training; 54% used a quantitative methodology, 18% qualitative, and 28% mixed methods. The quality of studies varied, with a greater proportion of low-quality studies (23%) and fewer high-quality studies (25%) reporting reactions than in the overall sample. Of the 74 papers, 73% reported wholly positive staff reactions to the training and 1% a predominantly negative learner response. Both positive and negative aspects of the training were reported in 22% of papers and 4% of studies compared two or more different approaches to training or education. A variety of methods for assessing reaction was used across the studies from informal discussions and semistructured interviews to questionnaires with open or fixed-response questions. These methods provided either a broad and sometimes blunt approach or a narrow and somewhat restrictive focus for learner feedback. In some cases, the restrictions of the method of gathering data limited the aspects of satisfaction learners were able to report, to those in which the trainer(s)/educator(s) were interested. However, other studies provided substantial detail on areas of the training that learners had found particularly beneficial or unhelpful. Therefore, due to some of the biases in questions asked, some caution has to be used when interpreting these results.

Four themes, drawn from 34 of the studies, of which the majority were moderate or high quality (88%), emerged from our CIS:

1. Relevance of training to learners’ role and practice
2. Teaching and learning approaches adopted
3. The quality of the training materials
4. Trainer/educator qualities

#### Relevance and Applicability to Role and Practice

The perceived applicability of training to a participant’s role and practice was an important factor in whether learners felt completion was beneficial. Studies indicated that training content, such as scenarios and case examples, must be relevant to the setting in which learners worked and to their role. Staff were unlikely to make efforts to apply learning in practice where training was perceived to lack relevance (Alnes, Kirkevold, & Skovdahl, 2013; Pereles et al., 2003). This finding supports assumptions within established models of adult learning (Knowles, 1970; Knowles, Holton, & Swanson, 2015; Taylor & Hamdy, 2013) and evidence from research on learning motivation in adult education more broadly (De Rijdt, Stes, van der Vleuten, & Dochy, 2013; Sogunro, 2015)

#### Teaching and Learning Approaches

Studies indicated that learners across a range of professional backgrounds, and at a range of levels, shared a preference for training that included interactive group work. Learners also indicated they valued training combining classroom-based theory with in-service/practice-based or experiential learning. Where e-learning was utilized, learners stated a combination of individual study with opportunities for on-line or face-to-face discussion were preferable. While the flexibility of e-learning was cited as a benefit, the requirements for specialist technical support and the time demands for learners and facilitators of engaging regularly with online discussions, which they felt were particularly beneficial to learning, meant this was suggested to be a resource intensive form of study, if done well.

A positive learning experience tended to be reported in studies employing written, video/DVD or roleplay-based case examples or vignettes, or through involving carers in delivery of training. They were particularly useful when they demonstrated good and poor practices for learners to reflect on and discuss. Opportunities within training to demonstrate and practice skills through in-service/practice-based learning were valued. However, the use of roleplay or simulated learning could be found distressing by learners, particularly where this was employed during an individual session where there had not been time to develop a trusting relationship between learners and facilitator.

#### Quality of Training Materials

Learners identified the quality of training materials as important. Higher quality materials were clear, easy to follow, informative and unambiguous, used straightforward language, and were concise. This was particularly important for learners who were learning in a second or additional language.

The perceived attractiveness of learning activities to learners has been highlighted within the theory of reasoned action, as an important antecedent for learners in their decision making process regarding intention to participate in workplace learning (Kyndt & Baert, 2013). Therefore, the relevance of the program, teaching and learning approaches used, quality of the materials, and feedback from peers who have already attended the training about their perceptions of these elements, may all impact on future uptake of offered training.

#### Trainer Skills and Qualities

Studies highlighted the need for a skilled trainer/facilitator. Trainer/facilitator qualities identified included the following: the ability to create a comfortable environment where learners were encouraged to ask questions; being knowledgeable about the topic; listening to participants; being able to tailor training to the individual needs of a group; being able to identify and address concerns and needs quickly; and responding positively and skillfully to challenge unhelpful learner views and attitudes. This finding adds to a growing body of research in workplace training and education, that not only should effective trainers/facilitators be experienced and skilled in facilitating training with adult learners (e.g., Gauld & Miller, 2004); a concept which has long since been established. It also supports evidence that trainers/facilitators should be able to use their own vocational knowledge and interpersonal skills to be “trainee-centered” (Arghode & Wang, 2016).

A number of authors have recently suggested that by using their knowledge of a relevant subject area, and by being able to tailor learning to a given learner group and their roles, trainers are in turn more effective (Arghode & Wang, 2016) and produce higher trainee satisfaction (Ghosh, Satawadi, Joshi, Ranjam, & Singh, 2012). This has especially been found to be the case in health care education (Khamarko, Koester, Bie, Baron, & Myers, 2012). Therefore, when combined with this growing body of evidence, our findings suggest that organizations (especially, but not limited to, health care organizations) could benefit from considering both previous facilitation experience and knowledge in the subject area, when recruiting or selecting trainers.

### Learning

Learning-related outcomes such as knowledge, attitudes, confidence, perceived competence, and self-efficacy were reported in 109 studies, 28 of which (26%) used mixed methods, 64 (59%) were quantitative studies, and the remaining 17 (16%) employed a qualitative design. Of these papers, 80% reported wholly positive outcomes for learning, 15% reported mixed outcomes with positive effects found in some areas and not in others and 6% reported no change in outcomes. The quality of the studies was largely moderate to good with only 14% rated as low quality, 55% as moderate quality, and 31% as high quality. Methods of data collection included validated measures, nonvalidated questionnaires, surveys or scales, interviews, focus groups, and analysis of documents such as assignments or written assessments. Of the 92 studies employing a mixed-methods or quantitative design, 51 (55%) used at least one validated measure of learning. While use of qualitative self-report or nonvalidated methods of assessing learning gains presents validity issues with regard to interpretation of findings, such data do offer a perspective on whether health and social care staff perceive they have learned anything. In some cases, staff may feel there have or have not been learning gains, whether or not this is seen on validated measures. This can have positive or negative impacts on motivation to change behavior or practice. However, some learning gains may be difficult to quantify and thus measure, for example, improved relationships with people with dementia or greater knowledge of individual life history and its use in day-to-day care. Therefore, while qualitative studies must be treated with caution in terms of their indication of learning gains, they retain value in understanding staff perceptions of changes in their knowledge, skills, and confidence to deliver complex dementia care, particularly in difficult to measure areas.

Our analysis revealed indicators associated with the efficacy of teaching methods for learning generally, which are discussed first. Three additional subcategories of components of learning were identified and analyzed separately: knowledge or understanding; attitudes and beliefs; and confidence, competence, and self-efficacy, which are discussed subsequently in this section.

#### Teaching Methods

While caution needs to be employed in interpreting the results, due to the variable study quality, predominantly positive findings across all studies and the low numbers of studies evaluating some teaching methods, the evidence suggested some teaching methods were consistently successful or unsuccessful. In-service/practice-based learning when used as the sole training approach, watching an individual or group DVD, and learning through reading a written resource (hard copy or electronic) were not effective in the majority of studies utilizing these approaches. The latter two approaches represent passive learning styles, and do not reflect the best practice, active learning approaches, advocated for in effective learning across the spectrum of education research (Darling-Hammond et al., 2008; Freeman et al., 2014; Jensen, Kummer, & Godoy, 2014; Mansouri & Lockyer, 2007; Pulsford, Jackson, O’Brien, Yates, & Duxbury, 2013; Zaher & Ratnapalan, 2012).

A small number of studies compared efficacy of different training approaches for learning, allowing comparison beyond a “no training” baseline or control. Generally, they found that active learning was more successful than passive approaches, for example, classroom and online multimedia methods were more effective than learning through reading written materials and multimedia online learning was more effective than passively watching a video lecture of the same content. However, in one study (Vollmar et al., 2010), no significant differences were found between learning through didactic lecture compared with multimedia online learning. There was a completion rate of only 50% for online learning, therefore, suggesting that passive face-to-face learning may be more useful by virtue of higher attendance from staff. Four studies compared face-to-face combined with in-service learning, with face-to-face learning alone (Resnick, Cayo, Galik, & Pretzer-Aboff, 2009) and in-service learning alone (Corwin, Owen, & Perry, 2008; McCabe et al., 2014; Smythe et al., 2014). All found in-service learning alone was less effective than either face-to-face delivery alone, or in-service learning in combination with face-to-face delivery. This indicates that theoretical content is required alongside, or to underpin, in-service learning, for effective learning to take place. In addition, the broader service-learning literature indicates that reflection forms an important component of in-service or practice-based learning, since it supports meaning making of the experience (Mitchell et al., 2015). None of the programmes using in-service learning explicitly utilized reflection as a component of this approach and this may account for some of the reduced efficacy of this teaching method.

#### Knowledge and Understanding

In 88 of the studies, outcomes related to improvements in knowledge were evaluated. Overwhelmingly (85%), studies reported staff increased their knowledge following training. In the 17% of studies adopting a qualitative methodology, 10 studies used staff self-report posttraining (via interviews, focus groups, or open-ended questionnaire) as the sole data collection method. All of these studies found staff felt attendance at training had increased their knowledge. Of the five studies also using observation of practice as a data collection method, two reported no change or mixed results for knowledge gains, suggesting this may offer a more robust method for assessing knowledge gains qualitatively than self-report alone. The remaining studies assessed knowledge gains using validated measures or nonvalidated questionnaires. Greater credibility has been given to studies using validated measures of knowledge within the synthesis, but all studies have contributed to the findings.

A training method that consistently led to no or weak knowledge gains was learning through a written resource (either hard copy or online), as assessed in three studies all using nonvalidated quantitative knowledge questionnaires, suggesting this should not be used as a primary mode of learning. This is a finding that is replicated in research in other health fields (Bluestone et al., 2013; Cervero & Gaines, 2014). A common feature of other studies reporting limited or no knowledge gains posttraining was utilization of in-service learning as the main mode of delivery, with two of the three studies using this method reporting variable, limited or no learning had taken place. The main reasons cited as barriers to effective in-service learning were, time available for the learner and mentor while working in-service, poor mentor engagement and a task-focused organizational culture (Skaalvik, Normann, & Henriksen, 2010; Skog, Negussie, & Grafstrom, 2000; Smythe et al., 2014). This indicates that it may be the poor setting conditions rather than in-service learning itself that is a barrier to learning via this method. However, of the studies reporting no knowledge gains, none had also evaluated learner reaction to training and, therefore, these issues were not explored systematically within the studies, to gain greater insight to them from a learner perspective.

Simulated, experiential, and roleplay-based learning produced weak or variable outcomes for knowledge gains in two out of the five studies adopting these approaches. A noticeable difference between the studies using these methods with positive knowledge gains and those with mixed or no gains, was the inclusion, in studies with positive results, of learner debriefing and feedback. This indicates it may be the way in which experiential/simulated learning was applied in these studies, rather than the method itself, which influenced learning efficacy. The existing body of research into simulated learning in nondementia education within health and social care indicates simulation can be highly effective in knowledge and skills development (Stocker, Burmester, & Allen, 2014). However, a critical component of learning within simulation, roleplay, or experiential situations lies within the structured debriefing process (Dedy, Bonrath, Zevin, & Grantcharov, 2013; Gjeraa, Møller, & Østergaard, 2014; Gordon, Darbyshire, & Baker, 2012; Kottewar, Bearelly, Bearelly, Johnson, & Fleming, 2014; Stocker et al., 2014), which appears to be missing from training involving simulation within a number of the dementia educational studies.

#### Attitudes and Beliefs

Thirty-three of the studies evaluated impact of training on staff attitudes or beliefs. Studies most commonly adopted a quantitative approach to measurement (81%), with the remaining studies adopting qualitative approaches including interviews, focus groups, and analysis of learners’ written assignments or reflective essays. Just over half (52%) of quantitative studies used a validated measure, with the remainder using a nonvalidated scale to assess attitude change. Of the 33 studies, 27% (*n* = 9) reported no significant change in attitude posttraining; one found improvements in attitudes for qualified nurses but not nondirect care staff (Long, 2013) and the remaining 23 studies (70%) reported positive attitude change. In four studies (Beville, 2002; R. Elliot & Adams, 2012; Mahendra, Fremont, & Dionne, 2013; van Zuilen et al., 2008), there were significant methodological weaknesses in the measures used to assess attitude change or in the data analysis techniques adopted and, therefore, their results were not used to identify issues of training efficacy in this component of analysis.

A feature of six of the eight studies, in which attitude change was not observed, was training delivery in one or more individual sessions of 2 hours or less duration, although multiple individual sessions were delivered to provide training programs that ranged from 2 to 12 hours in total. In contrast, 18 of the 23 studies (78%) reporting positive attitude change included individual training sessions of longer than 2 hours and were generally longer in overall program duration (total program lengths of 4 hours to 10 days); indicating that staff need consolidated time engaging with sessions of training in order to effect attitude change. A similar finding has been reported in other studies within health care services (Bjerrum, Tewes, & Pederson, 2012).

#### Confidence, Competence, and Self-Efficacy

Thirty-seven studies reported on the impact of training on staff confidence, competence, and self-efficacy in delivering dementia care. Over half (54%) adopted a quantitative methodology, 14% a qualitative design and 32% mixed methods. Studies were largely moderate (*n* = 23) and high quality (*n* = 10), with 5 of the 10 high-quality studies utilizing a validated measure of confidence, competence, or self-efficacy. In the remaining studies, confidence, competence, and self-efficacy were measured using a nonvalidated measure, questionnaire or survey, or through focus groups and interviews. Of the 37 studies, 81% reported improvements to staff confidence, competence, or self-efficacy, although significant positive change was found in only one-third of studies where this was measured using a validated tool (McCabe et al., 2014; Surr, Smith, Crossland, & Robins, 2016). Significant improvements were found in some subscales of competence, statistically but no clinically significant change was found in a further two (Elvish et al., 2014; Goyder, 2011) and no change in the remaining two (Finnema et al., 2005; Smythe et al., 2014). This suggests that nonvalidated measures may be unreliable in assessing changes in these areas. This supports findings in broader education research, which have consistently found self-report estimations of competency to be invalid (De Rijdt et al., 2013). However, despite these issues, the studies do provide information regarding whether health and social care staff feel they are more confident or competent in caring for people with dementia, whether or not this is evidenced through data collected using a validated measure. Staff reports of increased confidence, might in turn lead to additional positive outcomes, for example, improved role satisfaction or reduced stress and burden.

Twenty-six (70%) of the studies reporting improvements to staff confidence, competence, or self-efficacy utilized small or large group face-to-face learning, suggesting active participation and discussion appears important for these aspects of learning. The value of interaction in learning was also evidenced in the studies using e-learning. Studies evaluating interactive Web-based resources were largely found to increase confidence, competence, or self-efficacy, although in one study (Irvine, Beaty, Seeley, & Bourgeois, 2013), this effect was seen only for nonclinical staff. However, in a separate study, a written, noninteractive, Web-based resource did not increase general practitioner confidence in administering driving assessments to people with dementia (Moorhouse & Hamilton, 2014). Therefore, despite caution needing to be applied to results due to issues about methodological robustness of studies, (inter)active learning appears more effective than passive approaches, in supporting improved confidence or self-efficacy, a finding that replicates that found in other workplace learning fields (Bluestone et al., 2013).

Studies also indicated that a combination of theory (through classroom-based learning) and practice (in-service learning) was more likely to produce positive results in improving staff confidence, competence or self-efficacy. This finding also reflects those found in other fields of professional education (De Rijdt et al., 2013; Tolsgaard et al., 2014).

### Behavior Change

Sixty studies reported on changes to staff behavior or practices as an outcome of training. The majority of studies were rated as moderate (47%) or high quality (43%) with only 10% of low quality. Data collection methods included review of practice documents such as care records (40%), observation of staff behaviors and practice (27%), and staff self-report (40%), with some utilizing more than one method. Self-report can provide useful information about perceived behavior change, and may permit the exploration of the influence of potential confounding factors, thus providing contextual information about why behavior change may or may not have occurred. However, it is also open to potential bias. Nearly two fifths (37%) of studies relied solely on subjective staff self-report to assess behavior change and thus the results must be treated with caution, although 27% of these studies were rated as high quality and a further 64% moderate quality. Areas of change reported in studies included communication, antipsychotic prescribing and administration, person-centered care/general care practice improvements, restraint, and implementation of a specific care process or tool. Studies using self-report methods only were more likely to assess broader, less specific care improvements (e.g., delivery of care that is more person-centered, general practice changes) than studies using more objective measures. They were also more likely to report wholly or some positive outcomes for behavior change (86% and 14%, respectively), compared with studies using at least one objective measure (68% wholly positive, 26% mixed results, 5% no change on any outcomes).

The most commonly adopted teaching method in over half of studies (58%), where positive outcomes for behavior change were reported, was the inclusion of structured application of learning into practice. Approaches to achieving this included, in-practice activities or projects to be implemented as part of, between or after training sessions; expert clinical supervision; application of a participatory action research cycle; provision of tools or decision support software; and development of staff as Champions who support implementation of training in practice. Conversely, a common feature of over half (53%) of studies where mixed or no change outcomes were reported was use of a purely classroom-based approach to learning. While the content of this was varied and included active and passive approaches, application of learning in practice as part of the training itself was not present. This indicates the importance for behavior change, of supporting staff to implement learning in structured ways as part of training, ahead of expectations around application in everyday practice.

A further common feature of studies reporting positive behavior change was the inclusion of structured assessment or care planning tools, or care delivery approaches within training. This suggests staff may more readily adapt behaviors and practices when provided with a specific tool or method to guide change in a structured way. Overall, the importance of structured approaches to changing practice behaviors is indicated in the literature. The use of a structured tool or set process within dementia care training provides learners with a goal or desired behavior or way of practicing to be achieved. This finding is replicated in research conducted in other areas of education and training (Bluestone et al., 2013), which in particular identify goal setting as an effective method for driving behavior change (Goodwin, Ostuzzi, Khan, Hotopf, & Moss-Morris, 2016; Porcheret et al., 2014).

Of the 15 studies that reported mixed or no-change results for behavior change, only four also evaluated reaction and seven learning. Two of the studies that examined reaction reported mixed staff reactions, with lack of relevance/applicability of the training to practice being identified as a key issue by staff attending. This indicates staff may be less likely to make efforts to modify their behaviors or may be unable to do so, if the training does not provide them with relevant methods to support this. Of the studies also evaluating learning, six reported some improvements in knowledge and one improvement in staff confidence. One reported no significant change in staff attitudes. This suggests that while training may increase staff knowledge, this does not automatically translate into behavior change in practice.

### Outcomes, Results, or Impact

Fifty of the studies reported on the impact of education and training on outcomes or results, for people with dementia (76%), staff (32%), and family caregivers/relatives (8%). Studies tended to be of medium (46%) to high (50%) quality with only 4% rated as low quality. Only 8% utilized a qualitative design, with 82% adopting a quantitative methodology and 10% using mixed methods.

#### Outcomes for People With Dementia

Thirty-nine studies reported outcomes for people with dementia; however, in one study (Kellett, Moyle, McAllister, King, & Gallagher, 2010), data were collected solely via staff report using qualitative interviews, meaning it was excluded from the analysis due to unreliability of results due to potential bias. Most commonly, the outcomes evaluated in studies were impact on behaviors of people with dementia such as agitation, anxiety, and aggression (66%), quality of life (21%), depression (14%), communication (11%), and activities of daily living (11%).

In 68% of studies, a positive improvement in all or some outcomes was found and in all of these studies, the training adopted face-to-face teaching methods, with active participation for example through discussing examples from their own practice and engagement in problem solving forming a common feature. This finding is aligned with existing understanding regarding practice learning approaches most likely to lead to practice change: where situated learning, whereby learners collaboratively discuss genuine problems arising from their professional practice, provides optimal learning conditions (Webster-Wright, 2009). In a number of studies, this was supported by clinical supervision or in-service learning with mentorship, which offered learners the opportunity to gain feedback on and discuss their own practice. A similar finding is reported in broader in-service learning research, where clinically integrated teaching has been found to support improvements across a range of outcomes (Bluestone et al., 2013).

A second common feature was training facilitation by an experienced trainer who was usually also an experienced clinician. On the other hand, a feature of 30% of the training in studies where no change results were found was the use of a member of staff or in-house trainer to deliver some or all of the training intervention. Therefore, trainer qualities appear significant in influencing outcomes for people with dementia, beyond the level of participant reaction to training. While the current educational literature discusses areas such as instructor expertise and credibility with regard to student engagement, motivation (Imlawi, Gregg, & Karimi, 2015), satisfaction, and learning or educational outcomes (Paechter, Maier, & Macher, 2010), there is limited discussion of if and how instructor expertise affects the behavioral and outcome levels of the Kirkpatrick framework.

#### Outcomes for Family Members

Only four studies evaluated outcomes for family members/carers, in the form of satisfaction with care of their relative. In two of these, the training included a component focused on working positively with and engaging families. The two studies using qualitative methods reported greater satisfaction with care but also identified areas where families felt further improvements could be made. The two studies adopting quantitative measures of satisfaction found no significant changes between pre- and posttraining. Given the low numbers of studies in this area, it is not feasible to draw any further conclusions regarding training characteristics most likely to lead to improved family member outcomes.

#### Outcomes for Staff

Sixteen studies reported on outcomes related to staff, falling into three categories: job satisfaction and accomplishment (44%); stress, strain, and burden (56%); and exhaustion, burnout, and health complaints (38%) with some studies evaluating outcomes across multiple categories. Studies showed training was more likely to lead to no change than to positive outcomes across all categories. Studies that reported some positive outcomes for staff utilized training that was more likely to be longer in total duration (8+ hours in total) and comprised multiple individual sessions of 90 minutes to a full-day duration. Studies reporting no change results utilized training which was more likely to be shorter overall (less than 7.5 hours) and have shorter individual session length (1 hour or less). This suggests that increasing staff job satisfaction and reducing stress or exhaustion is more likely to occur if training permits greater depth of staff engagement in terms of the overall training program, and individual session length. This finding mirrors that of the broader professional development literature, which advocates an educational model of ongoing, situated learning within a workplace context, as opposed to a “training” model of discrete sessions, decontextualized from real-world practice (Webster-Wright, 2009). Given the earlier finding that staff attitudes are more likely to be improved in studies with longer individual session duration, this suggests there may be some interaction between staff attitudes toward people with dementia and their feelings of role satisfaction, stress, or exhaustion, a finding supported by research in this area (Brodaty, Draper, & Low, 2003; Willemse et al., 2014). This suggests some interrelationship is likely to be present between the Kirkpatrick levels, for staff-related outcomes.

A further feature of over half of the studies reporting positive staff outcomes was inclusion in the training of a structured tool, manual, or practice guidelines. This suggests that staff may feel more satisfied and less stressed, if they are provided with materials that help give a clear structure to follow in their practice.

### Studies Evaluating Impact Across All Levels

Only three studies evaluated all levels of the framework (Clare et al., 2013; McCurry, LaFazia, Pike, Logsdon, & Teri, 2012; Speziale, Black, Coatsworth-Puspoky, Ross, & O’Regan, 2009). Two of these studies were high-quality and one moderate and all used a quantitative design. The training programs they utilized were similar in delivery methods (classroom and classroom + practice-based work), were of at least 3 hours total duration, and had individual session length of 1 to 2 hours (where reported). All reported positive learner reactions to the training, improvements in knowledge posttraining, improvements in behavior posttraining and at least some improved outcomes for people with dementia. However, none of the studies discussed the interrelationship between factors at each Kirkpatrick level in contributing to efficacy of the training program and their quantitative design limits their ability to offer learner or service-based explanations for potential efficacy.

## Discussion

The review revealed a wide range of published international research, evaluating efficacy of dementia training for staff working across a wide range of health and social care settings. As was found in previous systematic literature reviews in this area (Alushi et al., 2015; Beeber et al., 2010; Brody & Galvin, 2013; Eggenberger et al., 2013; K. E. J. Elliott et al., 2012; Fossey et al., 2014; Kuske et al., 2007; McCabe et al., 2007; Perry et al., 2011; Raymond et al., 2014; Spector et al., 2013; Zientz et al., 2007), both the quality of evidence and reported efficacy of training interventions varied. This review identified that research has predominantly been conducted in care home settings, largely with qualified nurses or nurse aides/care assistants. There were smaller numbers of studies in other health and social care settings and with other staff groups. Existing research has evaluated training delivered using a variety of teaching methods, although predominantly face-to-face learning was adopted often alongside other methods. However, a limitation of many of the studies was use of a pre–post design, or a “no training” control group in randomized studies. Few studies compared the efficacy of different training methods against each other. Therefore, where positive results were found, this only provided an indicator of efficacy compared with receiving no training and thus provided limited understanding of whether the methods used were optimal for delivering particular outcomes. There was a general lack of attempt to address potential methodological bias in the majority of studies, with many using qualitative self-report and nonvalidated measures or questionnaires to assess change on outcomes, sometimes only collected immediately posttraining, meaning comparison with pretraining or assessing impact on longer term practice were not possible. On the other hand, studies using validated measures often did not collect additional data; therefore, failing to examine what learners felt were particularly useful or unhelpful components of training that could help explain quantitative results. A further limitation of the majority of studies was their focus on only one or two Kirkpatrick levels of efficacy, meaning they were unable to examine potential interrelationships between factors. Only three studies evaluated all levels of the framework (Clare et al., 2013; McCurry et al., 2012; Speziale et al., 2009), but none has explored the interrelationship between factors. We, therefore, have provided some discussion of the potential interrelationships across Kirkpatrick levels, based on our synthesis of the evidence and inferences we can draw from studies evaluating two or more levels. However, our conclusions remain tentative given the lack of exploration of these relationships within published studies. Therefore, as with previous systematic reviews, this study indicates the need to conduct further robust research that examines the efficacy of dementia training, taking into account the multifaceted range of outcomes, and interrelationships between factors that are likely to influence these. The use of mixed methods, which can provide robust evidence of efficacy, but also the possibility to explain findings through gaining learner and facilitator perspectives, would appear advantageous. Future research should also engage more clearly with existing education research in workplace learning across other professions. While there are some factors which may be particular to a specific professional workforce, such as that providing dementia care, our review has identified similarities of experience across education research of different workforce groups and thus suggests benefits to dementia educators of engaging with this body of literature.

Despite the limitations of existing research, this review has identified a number of key features that seem to exist in effective dementia training and which support understanding of approaches to effective professional development and workplace education more broadly. Training/education most likely to be effective:

- Is relevant and realistic to the role, experience, and practice of learners rather than a one-size-fits-all training program
- Includes active participation
- Underpins practice-based learning with theoretical or knowledge-based content
- Ensures experiential and simulation-based learning includes adequate time for debriefing and discussion
- Is delivered by an experienced trainer/facilitator who is able to adapt it to the needs of each group
- Does not involve reading written materials (paper or Web-based) or in-service learning as the sole teaching method
- Is of a total duration of 8+ hours with individual training sessions of at least 90 minutes
- Includes active, small, or large group face-to-face learning either alone or in addition to another learning approach
- Includes learning activities that support the application of training into practice
- Provides staff with a structured tool, method or practice guideline to underpin care practice

These features will be useful to consider in designing future dementia training and educational programs. Given the similarities within our findings, to those reported in other areas of adult and workplace education, they may also provide the basis for more general principles to inform the design and delivery of adult and workplace education and professional development, across health and social care and in other professional sectors such as teaching and business.

### Limitations

Limitations to this review are that the inclusion only of articles published in English since 2000 may have excluded non-English language and older studies that might further contribute to understanding of effective dementia training and education. While using CIS and open inclusion criteria has permitted a broad examination of the evidence base, caution is however needed in interpreting the results. The limited number of studies in settings other than care homes and hospitals means application of findings to other settings providing dementia care, such as community and primary care requires caution.

### Conclusion

Despite methodological weaknesses and variability in methods adopted, there are some common features of training/education programs that appear more efficacious and these may be adopted as underpinning guidelines for the design of new dementia training and educational programs. They may also have relevance for adult professional development and workplace learning across a broad range of workplace settings. However, further robust research on the ingredients that lead to efficacy of dementia education and training is urgently required, to avoid continued utilization of programs that may have limited positive benefits.

## Supplementary Material

Supplementary material

Supplementary material

## Notes

We thank Frances Darby, Raji Thannivila Rajan, and Sarah Asquith for their help with screening and data extraction. This is independent research supported by the Department of Health Policy Research Programme (Understanding Effective Dementia Workforce Education and Training: A Formative Evaluation [DeWET Evaluation]; PR-R10-0514-12006). The views expressed in this article are those of the authors and not necessarily those of the Department of Health. The research was commissioned on behalf of Health Education England (HEE).
