# Supplementary material for: Effective Dementia Education and Training for the Health and Social Care Workforce: A Systematic Review of the Literature
Source: Rev Educ Res. 2017 Jul 31;87(5):966–1002. doi: 10.3102/0034654317723305 (PMC5613811; doi:10.3102/0034654317723305)
Supplement: Supplementary material [file Table_S1_Included_studies_online_supp.docx]

Table S1:

*Included studies*

| **Authors** | **Title** | **Year** | **Journal** | **Research aims** | **Country** | **Setting** | **Participants** | **Research methodology** |
| --- | --- | --- | --- | --- | --- | --- | --- | --- |
| Alnes, Kirkevold & Skovdahl | Insights gained through Marte Meo counselling: experiences of nurses in dementia specific care units. | 2011 | International Journal of Older People Nursing | To uncover what nurses perceived to have learned, during their participation in video supported counselling, based on Marte Meo principles, in four dementia specific care units | Norway | Care Homes | 26 registered nurses (RNs), enrolled nurses (ENs) and nurse aids (NAs) | Qualitative |
| Alnes, Kirkevold & Skovdahl | The influence of the learning climate on learning outcomes from Marte Meo counselling in dementia care | 2013 | Journal of Nursing Management | To identify factors that affected the learning outcomes from Matte Meo counselling | Norway | Care Homes | 26 Registered Nurses, Enrolled Nurses and Nursing assistants | Qualitative |
| Amos, Bush, Byszewski, Dalziel, Graham, Guzman, Hunt, Man-Son-Hing & Marshall | A continuing medical education initiative for canadian primary care physicians: the driving and dementia toolkit | 2003 | Journal of the American Geriatrics Society | To evaluate effects of the Driving and Dementia Toolkit on physician knowledge, confidence gained and usefulness | Canada | Primary Care | 86 Primary care physicians | Quantitative |
| Arcand, Monette, Monette, Sourial, Fournier, Gore & Bergman | Educating Nursing Home Staff About the Progression of Dementia and the Comfort Care Option: Impact on Family Satisfaction with End-of-Life Care | 2009 | JAMDA | The objective of this study was to assess the impact, in terms of family satisfaction with end-of-life care, of a nursing home (NH) pilot educational program for nursing staff and physicians on comfort care and advanced dementia. | Canada | Care Homes | Care home staff, 58 family members | Qualitative, |
| Asthill | Staff training and challenging behaviour in a day hospital | 2004 | Dementia | To evaluate a staff training intervention designed to facilitate a sustainable impact on care practice as measured by changes in behaviour found to be challenging | UK | Day Care | 7 Staff members | Quantitative |
| Barbosa, Nolan, Sousa & Figueiredo | Supporting direct care workers in dementia care: effects of a psychoeducational intervention | 2015 | American Journal of Alzheimer's Disease and Other Dementias | Assessing effects of a person-centred care-based psychoeducational intervention on care workers' stress, burnout and job satisfaction | Portugal | Care Homes | 58 Direct care workers | Mixed methods |
| Beer, Horner, Flicker, Scherer, Lautenschlager, Bretland, Flett, Schaper & Almeida | A Cluster-Randomised Trial of Staff Education to Improve the Quality of Life of People with Dementia Living in Residential Care: The Direct Study | 2011 | PlosOne | To determine if the delivery of education designed to meet the perceived need of GPs and care staff improves the quality of life of participants with dementia living in residential care. | Australia | Care Homes | 351 residents 55 GPs, 292 next of kin informants and 326 Residential Aged care facility staff. | Quantitative |
| Beer, Lowry, Horner, Almeida, Scherer, Lautenschlager, Bretland, Flett, Schaper & Flicker | Development and evaluation of an educational intervention for general practitioners and staff caring for people with dementia living in residential facilities | 2011 | International Psychogeriatrics | This study aimed to develop educational interventions [in dementia] for GPs and RCF staff tailored to meet their perceived educational needs. | Australia | Care Homes + Primary Care | 16 GPs, 18 residential care facilities and 326 staff. | Quantitative |
| Beer, Hutchinson & Skala-Cordes | Communicating With Patients Who Have Advanced Dementia: Training Nurse Aide Students | 2012 | Gerontology & Geriatrics Education | To examine if and how training NA students in communicating with patients who have advanced dementia changed their perceptions on quality of care and ability to communicate with these patients. | USA | Higher Education | 47 nursing aide students | Quantitative |
| Beville | Virtual Dementia Tour Helps Sensitize Health Care Providers | 2002 | American Journal of Alzheimer’s Disease and Other Dementias | A controlled simulation of degenerative physical symptoms common in dementia, such as impaired vision and motor skills, to give staff a broader sense of the patient’s perspective. | USA | All | 146 Staff from various 'elder care' settings e.g CNAs, Social Workers/activity directors, licensed staff and directors. | Quantitative |
| Bluethmann | Enhancing Quality Through Staff Training | 2008 | Alzheimer’s Care Today | Discusses the Alzheimer’s Association Foundations of Dementia Care training curriculum, including the results of field testing with residential care professionals. | USA | Care Homes + Assisted Living | 71 care home staff (Phase 1), 52 care home staff (Phase 2) | Mixed methods |
| Bourgeois, Dijkstra, Burgio & Allen | Communication Skills Training for Nursing Aides of Residents with Dementia: The Impact of Measuring Performance | 2004 | Clinical Gerontologist | To increase the quantity and quality of nursing aides' verbal interactions with clients with dementia during care routines | USA | Care Homes | 23 licensed nurses, 126 nursing aides, 125 residents with dementia | Quantitative |
| Brooker, Latham, Evans, Jacobson, Perry, Bray, Ballard, Fossey, & Pickett | FITS into practice: translating research into practice in reducing the use of anti-psychotic medication for people with dementia living in care homes | 2015 | Aging & Mental Health | To report on the acceptability and effectiveness of the FITS (Focussed intervention training and support) into practice programme - an intervention scaled up from an earlier cluster RCT that had proven successful in significantly decreasing antipsychotic prescribing in care homes. | UK | Care Homes | 66 Dementia care coaches | Mixed |
| Broughton, Smith, Baker, Angwin, Pachana, Copland, Humphreys, Gallois, Byrne & Chenery | Evaluation of a caregiver education program to support memory and communication in dementia: A controlled pretest–posttest study with nursing home staff | 2011 | International Journal of Nursing Studies | To evaluate the effects of the RECAPS and MESSAGE DVD based training on knowledge of support strategies and caregiver satisfaction in nursing home care staff and evaluate staff opinion of the training | Australia | Care Homes | 68 staff including nursing assistants, qualified nurses and recreational activities officers | Quantitative |
| Brown Wilson, Swarbrick, Pilling, & Keady | The senses in practice: enhancing the quality of care for residents with dementia in care homes | 2013 | Journal of Advanced Nursing | To develop, deliver and evaluate a training programme in care homes to enhance the quality of care for people living with dementia based on the principles of relationship-centred care expressed through the Senses Framework. | UK | Care Homes | 11 staff members, 11 residents | Qualitative |
| Burgess & Page | Educating nursing staff involved in the provision of dementia care | 2003 | Nursing Times | To measure the effectiveness of the nurse educator in dementia (G grade nursing post) | UK | Hospital | 172 staff members, 52 relatives of patients with dementia | Qualitative |
| Burgio, Allen-Burge, Roth, Bourgeois, Dijkstra, Gerstle, Jackson & Bankster | Come Talk With Me: Improving Communication Between Nursing Assistants and Nursing Home Residents During Care Routines | 2001 | The Gerontologist | To examine the efficacy of communication skills training and the use of individualized memory books in improving communication between CNAs and a larger group of residents during care. | USA | Care Homes | 64 Certified Nursing Assistants, and 67 Residents | Quantitative |
| Burgio, Stevens, Burgio, Roth, Paul & Gerstle | Teaching and Maintaining Behavior Management Skills in the Nursing Home | 2002 | The Gerontologist | To examine the efficacy of a comprehensive behaviour management skills training program for improving certified nursing assistants’ skill performance in the nursing home, to assess the effectiveness of a staff motivational system for maintaining newly acquired behaviour management skills for a 6-month period, and to evaluate any resulting effects on resident agitation. | USA | Care Homes | 85 Certified Nursing Assistants, 88 nursing home residents | Quantitative |
| Cameron, Horst, Lawhorne & Lichtenberg | Evaluation of academic detailing for primary care physician dementia education | 2010 | American Journal of Alzheimer's Disease and Other Dementias | To assess the effect of academic detailing as a strategy to increase early detection of dementia in primary care practice and to improve support and management of Alzheimer's disease and other dementias by increasing communication and referrals to local community agencies. | USA | Primary Care | 104 Physicians, 248 office/clinic staff | Quantitative |
| Cartwright, Franklin, Forman & Freegard | Promoting collaborative dementia care via online interprofessional collaboration | 2013 | Australasian Journal on Ageing | To develop, implement and evaluate an online interprofessional education dementia case study for health science students. | Australia | Higher Education | 125 students across the 5 health sciences disciplines | Mixed methods |
| Chang & Lin | Effects of a feeding skills training programme on nursing assistants and dementia patients | 2005 | Journal of Clinical Nursing | To develop a comprehensive feeding skills training programme for nursing assistants and to test the effects of this training programme on their knowledge, attitude and behaviour and the outcome of dementia patients including total eating time, food intake and feeding difficulty. | Taiwan | Care Homes | 20 nursing assistants  20 residents with dementia (12 in the treatment; eight in the control) | Quantitative |
| Chang, Wykle & Madigan | The Effect of A Feeding Skills Training Program for Nursing Assistants Who Feed Dementia Patients in Taiwanese Nursing Homes | 2006 | Geriatric Nursing | To assess if nursing assistants who completed the feeding skills training program have more knowledge, a more positive attitude, better perceived behavior control, greater intention, and better behavior in assisting residents with dementia to eat than those who did not complete the program. | Taiwan | Care Homes | 67 nursing assistants (treatment, n=31; control, n=36).  36 nursing assistant/resident with dementia dyads (treatment n = 20; control = 16) | Quantitative |
| Chenoweth, King, Jeon, Brodaty, Stein-Parbury, Norman, Haas & Luscombe | Caring for Aged Dementia Care Resident Study (CADRES) of person-centred care, dementia-care mapping, and usual care in dementia: a cluster-randomised trial | 2009 | Lancet Neurology | A randomised comparison of person-centred care, dementia-care mapping, and usual care. | Australia | Care Homes | Care staff, 289 residents | Quantitative |
| Clare, Whitaker, Woods, Quinn, Jelley, Hoare, Woods, Downs & Wilson | AwareCare: a pilot randomized controlled trial of an awareness-based staff training intervention to improve quality of life for residents with severe dementia in long-term care settings. | 2013 | International Psychogeriatrics | To establish whether training care staff to observe and identify signs of awareness in residents with severe dementia resulted in improved quality of life for residents. | UK | Care Homes | 65 Care staff, 65 Residents | Quantitative |
| Coogle, Head & Parham | Person-centred care and the workforce crisis: a statewide professional development initiative. | 2004 | Educational Gerontology | Implementation of a collaborative dementia training program to promote a greater understanding of person-centered care techniques, with the ultimate goal of improving the quality of care given to people with dementia. | USA | All | 71 Trainers, 832 Trainees: Nursing assistants, licensed practical nurses and registered nurses. | Quantitative |
| Coogle, Head & Parham | The long-term care workforce crisis: Dementia-care training influences on job satisfaction and career commitment | 2006 | Educational Gerontology | To compare changes in job satisfaction and career commitment among Alzheimer's care staff participating in a two-phase, state-level training collaborative to improve dementia care. | USA | All | 53 trainees - nursing assistants, licensed practical nurses and registered nurses | Quantitative |
| Cooke, Moyle, Venturato, Walters & Kinnane | Evaluation of an education intervention to implement a capability model of dementia care | 2014 | Dementia | To outline an education protocol used for carers as an intervention in a project that implemented and evaluated a capability model of dementia care (CMDC) in three long term aged care facilities in Queensland, Australia. | Australia | Care Homes | 48 staff in 3 care homes | Mixed methods |
| Corwin, Owen & Perry | Student service learning and dementia: bridging classroom and clinical experiences | 2008 | Journal of Allied Health | To find out whether students in Speech-Language Pathology and Nursing perceive benefits from participating in a service learning project involving residents of a long-term care facility who had dementia. | USA | Higher Education | 53 students and 19 Individuals with dementia. | Quantitative |
| Dalsgaard, Kallerup & Rosendal | Outreach visits to improve dementia care in general practice: a qualitative study | 2007 | International Journal for Quality in Health Care | To describe the outcome of a facilitator programme from the GPs' perspective and to explore how the communication between facilitators and GPs contributes to these outcomes | Denmark | Primary Care | 4 facilitators (GPs), 19 GPs | Qualitative |
| Davies, Lambert, Turner, Jenkins, Aston & Rolfe | Making a difference: using action research to explore our educational practice | 2014 | Educational Action Research | To conduct an action research evaluation of a dementia Care Training package | UK | Higher Education | 23 staff from care homes | Qualitative |
| Davison, McCabe, Visser, Hudgson, Buchanan & George | Controlled trial of dementia training with a peer support group for aged care staff | 2007 | International journal of geriatric psychiatry | To evaluate the impact of an eight-session training program for aged care staff in managing dementia-related challenging behaviours. | Australia | Care Homes + Assisted Living | 44 registered nurses, 46 nursing assistants. 113 residents with dementia. | Quantitative |
| Deudon, Maubourguet, Gervais, Leone, Brocker, Carcaillon, Riff, Lavallar & Robert | Non- pharmacological management of behavioural symptoms in nursing homes | 2009 | International journal of geriatric psychiatry | To evaluate the effectiveness of a staff education intervention to manage BPSD in older people with a diagnosis of dementia. | France | Care Homes | 306 residents; numbers participating in training not disclosed. | Quantitative |
| Downs, Turner, Bryans, Wilcock, Keady, Levin, O'Carroll, Howie & Iliffe | Effectiveness of educational interventions in improving detection and management of dementia in primary care: cluster randomised controlled study | 2006 | British Medical Journal | To test the effectiveness of educational interventions in improving detection rates and management of dementia in primary care | UK | Primary Care | GPs. Records of 450 eligible patients used. | Quantitative |
| Edvardsson, Sandman & Borell | Implementing national guidelines for person-centered care of people with dementia in residential aged care: effects on perceived person-centeredness, staff strain, and stress of conscience | 2014 | International Psychogeriatrics | This study aimed to evaluate the effects of implementing national guidelines for person-centred care of people with dementia on self-reported person-centeredness, strain, and stress of conscience as perceived by care staff. | Sweden | Care Homes | 171 staff members | Quantitative |
| Edwards, Voss & Iliffe | The development and evaluation of an educational intervention for primary care promoting person-centred responses to dementia | 2013 | Dementia | This work aimed to develop and evaluate an educational intervention for primary care promoting person-centred responses to people experiencing cognitive decline. | UK | Primary Care | 94 primary care staff | Quantitative |
| Elliott & Adams | Using a practice development project to improve standards of care for people with dementia | 2012 | Nursing Older People | To report on the results of a training, education and development project for older people (CAMTED-OP) set up by Cambridgeshire and Peterborough NHS Foundation Trust to work with paid carers of people with dementia in care home settings. | UK | Care Homes | 86 Care home staff | Quantitative |
| Ellis | Carer-driven dementia education for professionals | 2008 | Nursing Older People | To report on a pilot project taking an action research approach to provide dementia awareness education for nurses on acute wards. | UK | Hospital | 97 Nurses | Mixed |
| Elvish, Burrow, Cawley, Harney, Graham, Pilling, Gregory, Roach, Fossey & Keady | ‘Getting to Know Me’: the development and evaluation of a training programme for enhancing skills in the care of people with dementia in general hospital settings | 2014 | Aging & Mental Health | To report on the development and evaluation of a staff training intervention in dementia care designed for use in the general hospital setting: the ‘Getting to Know Me’ training programme. To undertake initial psychometric analysis on two new outcome scales designed to measure knowledge and confidence in dementia care. | UK | Hospital | 71 general hospital staff | Quantitative |
| Emerson Lombardo, Wu, Hohnstein & Chang | Chinese Dementia Specialist Education Program: Training Chinese American Health Care Professionals as Dementia Experts | 2002 | Home Health Care Services Quarterly | To evaluate a Chinese Dementia Specialist Education Program (CDSEP). | USA | Community | 22 health and social care staff | Quantitative |
| Engelman, Altus, Mosier and Mathews | Brief training to promote the use of less intrusive prompts by nursing assistants in a dementia care unit | 2003 | Journal of Applied Behavior Analysis | To evaluate the use of simplified, brief caregiver training to promote the use of the System of Least Prompts (SLP) procedure and to help older adults with dementia to maintain independence. | USA | Assisted living | 2 care assistants and 3 residents | Quantitative |
| Ervin & Koschel | Dementia care mapping as a tool for person centred care | 2012 | Australian Nursing Journal | To embed person centred practices in rural residential aged care facilities through staff training in dementia care mapping (DCM). | Australia | Care Homes | 12 care home staff members | Quantitative. |
| Featherstone, James, Powell, Milne & Maddison | A controlled evaluation of a training course for staff who work with people with dementia | 2004 | Dementia | To increase staff’s insight relating to ‘the experience of having dementia’, via improving their knowledge-bases, and to use this new insight to improve attitudes and staff coping strategies. | UK | Care Homes | 40 care workers | Quantitative |
| Figueiredo, Barbosa, Cruz, Marques & Sousa | Empowering Staff in Dementia Long-Term Care: Towards a More Supportive Approach to Interventions | 2013 | Educational Gerontology | To assess a psychoeducational program for staff in care homes. The program was designed to increase knowledge regarding dementia care, promote skills to integrate motor and multisensory stimulation in daily care, and develop coping strategies to manage emotional workrelated demands. | Portugal | Care Homes | 6 care home staff, 6 residents | Qualitative |
| Finnema, de Lange, Droes, Ribbe & van Tilburg | The quality of nursing home care: do the opinions of family members change after implementation of emotion-oriented care? | 2001 | Journal of Advanced Nursing | To find out whether the application of integrated emotion-oriented care influences how the relatives of a person with dementia judge the quality of care offered | Netherlands | Care Homes | 230 staff; 194 residents and their families | Quantitative |
| Finnema, Droes, Erttema, Ooms, Ader, Ribbe & van Tilburg | The effect of integrated emotion-oriented care versus usual care on elderly persons with dementia in the nursing home and on nursing assistants: a randomized clinical trial | 2005 | International Journal of Geriatric Psychiatry | To examine the effect of integrated emotion-oriented care, on nursing home residents with dementia and nursing assistants. | Netherlands | Care Homes | 99 nursing assistants, 146 residents with dementia | Quantitative |
| Fossey, Ballard, Juszczak, James, Alder, Jacoby & Howard | Effect of enhanced psychosocial care on antipsychotic use in nursing home residents with severe dementia: cluster randomised trial | 2006 | BMJ | To evaluate the effectiveness of a training and support intervention for nursing home staff in reducing the proportion of residents with dementia who are prescribed neuroleptics. | UK | Care Homes | Care home staff, 168 care home residents and 181 family members | Mixed method |
| Frade | Developing a learning set within dementia care: A practice development project | 2005 | Dementia | To promote staff learning within wards providing dementia care | UK | Hospital | Dementia care nurses | Qualitative |
| Fruhauf, Jarrott & Lambert-Shute | Service-Learners at Dementia Care Programs: An Intervention for Improving Contact, Comfort, and Attitudes | 2004 | Gerontology & Geriatrics Education | To report on the results of a training program designed to facilitate comfort with older adults who have dementia for service-learners in a gerontology course serving at an Adult Day Services (ADS) program. | USA | Higher Education | 16 Undergraduate students | Quantitative |
| Galvin, Kuntemeier, Al-Hammadi, Germino, Murphy-White & McGillick | “Dementia-friendly hospitals: care not crisis” an educational program designed to improve the care of the hospitalized patient with dementia | 2010 | Alzheimer Disease and Associated Disorders | The development, implementation and evaluation of a program entitled “Dementia-Friendly Hospitals: Care Not Crisis.” | USA | Hospital | 548 hospital staff from 6 hospitals | Quantitative |
| Goyder | Staff Training using STAR (Staff Training in Assisted Living Residences): A Pilot Study in UK Residential Care Homes (PART 2) | 2011 | PhD Thesis | A focus on the use of staff training intervention to reduce the Behavioural and Psychological Symptoms of Dementia (BPSD) in residents living in care homes. An empirical study designed to evaluate the feasibility of delivering the STAR programme to staff in UK care homes, and to evaluate the effects on BPSD in residents with dementia and the influence on staff attitudes and competency. | UK | Care Homes | 25 non-qualified care staff, 29 residents | Mixed methods |
| Gozalo, Prakash, Qato, Sloane & Mor | Effect of the Bathing Without a Battle Training Intervention on Bathing-Associated Physical and Verbal Outcomes in Nursing Home Residents with Dementia: A Randomized Crossover Diffusion Study | 2014 | Journal of the American Geriatrics Society | To evaluate the effectiveness of the Bathing Without a Battle intervention in reducing physical and verbal aggressive behaviors for nursing home residents with dementia. | USA | Care Homes | All staff in 6 nursing homes, 240 Nursing home residents with dementia | Quantitative |
| Hobday, Savich & Gaugler | An Internet-Based Multimedia Education Prototype to Enhance Late-Stage Dementia Care: Formative Research Results | 2010 | Geriatric Nursing | To develop a portable, Internet-based multimedia education program (IBME) to provide a more efficient training resource for direct care workers (DCWs) who care for nursing home residents suffering from late-stage dementia | USA | Care Homes | 34 direct care staff | Mixed methods |
| Hobday, Savik, Smith & Gaugler | Feasibility of Internet Training for Care Staff of Residents with Dementia: The CARES® Program | 2010 | Journal of Gerontological Nursing | To evaluate the ability of CARES® (a web-based, interactive, multimedia educational program for professional and paraprofessional caregivers in nursing homes) to improve certified nurse assistants' knowledge and perceptions of competency, communication, and gain related to dementia care in two types of residential settings. | USA | Care Homes + Assisted Living | 40 Nursing Assistants | Quantitative |
| Innes | Student-Centred learning and Person-centred dementia care. | 2001 | Education and ageing | Evaluation of a person-centred dementia care education programme where theories of adult learning and student-centred learning were utilised. | UK | Care Homes | 100 staff members: nurses, care assistants, domestic staff and managers | Mixed methods. |
| Innes, MacKay & McCabe | Dementia studies online: reflections on the opportunities and drawbacks of eLearning | 2006 | Journal of Vocational Education and Training | To explore the opportunities and drawbacks of eLearning from the viewpoints of educators and learners. | UK | Higher Education | 3 tutors, 29 past and current students | Mixed methods |
| Irvine, Ary & Bourgeois | An Interactive Multimedia Program to Train Professional Caregivers | 2003 | Journal of Applied Gerontology | To develop and evaluate a staff training program using the interactive multimedia technology (IMM) format. | USA | Care Homes | 88 professional caregivers | Quantitative |
| Irvine, Beaty, Seeley & Bourgeois | Use of a Dementia Training Designed for Nurse Aides to Train Other Staff | 2012 | Journal of Applied Gerontology | To provide non-direct care workers (NDCWs) with skills training to interact with residents with dementia. | USA | Care Homes | 68 non-direct care workers | Quantitative |
| Isaacson, Safdieh & Ochner | Effectiveness of a modified Continuum curriculum for medical students: A randomized trial | 2011 | Neurology | To test the effectiveness of a modified version of the Dementia module of the Continuum curriculum, adapted for medical students rotating on their neurology clerkship, in increasing medical knowledge of dementia. | USA | Higher Education | 226 third and fourth year medical students | Quantitative |
| Jefferson, Cantwell, Byerly & Morhardt | Medical student education program in Alzheimer’s disease: The PAIRS Program | 2012 | BMC Medical Education | To evaluate the PAIRS Program and its effectiveness in enhancing medical education as a service-learning activity and replication model for the Buddy Program^TM^. | USA | Higher Education | 45 first year medical students | Mixed methods |
| Jeon, Luscombe, Chenoweth, Stein-Parbury, Brodaty, King & Haas | Staff outcomes from the Caring for Aged Dementia Care REsident Study (CADRES): A cluster randomised trial | 2012 | International Journal of Nursing Studies | To examine the impact of person centred care and dementia care mapping compared to each other and to usual dementia care on staff outcomes, in terms of staff burnout, general well-being, attitudes and reactions towards resident behavioural disturbances, perceived managerial support, and quality of care interactions. | Australia | Care Homes | 194 managers, nurses, therapists and nurse assistants working in the participating sites | Quantitative |
| Juola, Bjorkman, Pylkkanen, Finne-Soveri, Soini Kautiainen, Bell & Pitkala | Feasibility and baseline findings of an educational intervention in a randomized trial to optimize drug treatment among residents in assisted living facilities | 2014 | European Geriatric Medicine | To describe the baseline findings and feasibility of a cluster randomized controlled trial of staff training to optimize the use of drugs among older residents in assisted living facilities. | Finland | Assisted Living | 17 members of nursing staff, 3 consultants, 227 residents | Quantitative |
| Kaf, Barboa, Fisher & Snavely | Effect of Interdisciplinary Service Learning Experience for Audiology and SpeechLanguage Pathology Students Working With Adults With Dementia | 2011 | American Journal of Audiology | To examine changes in audiology and speech-language pathology students’ attitudes toward adults with dementia following an interdisciplinary service learning (SL) experience in which they socialized with nursing home residents who had dementia. | USA | Care Homes | 75 Audiology and speech-language pathology students, 24 residents | Mixed methods |
| Kalsy, Heath, Adams & Oliver | Effects of training on controllability attributions of behavioural excesses and deficits shown by adults with Down syndrome and dementia | 2007 | Journal of Applied Research in Intellectual Disability | To examine the effects of care staff training in ageing, dementia and people with intellectual disabilities on the attributional style (specifically controllability) and optimism for change in behavioural deficits and excesses. | UK | Day care | 97 care staff | Quantitative |
| Kellett, Moyle, McAllister, King & Gallagher | Life stories and biography: a means of connecting family and staff to people with dementia | 2010 | Journal of Clinical Nursing | To assess the impact of newfound biography knowledge on family–staff caregiver attitudes, perceptions of roles, conflict and the subsequent management of stress using participatory dementia care practices | Australia | Care Homes | 7 family care-givers, 7 staff members | Qualitative |
| Kelly | Changes in knowledge and attitudes of certified nursing assistants about ethics of treatment choices for nursing home residents with end-stage Alzheimer’s disease | 2004 | PhD Thesis | To measure and analyse the change in CNAs’ knowledge about ethics of treatment decisions and attitudes about treatment choices for SNF residents with end-stage AD after the implementation of an education intervention. To determine if added support sessions make a difference in the degree of change in knowledge and attitudes compared to those who receive only the education intervention. | USA | Care Homes | 60 Certified Nursing Assistants (CNAs) | Quantitative |
| Kemeny, Boettcher, DeShon & Stevens | Using Experiential Techniques for Staff Development: Liking, Learning, and Doing | 2006 | Journal of Gerontological Nursing | To evaluate whether the role-playing techniques used in an Alzheimer’s disease research project are effective in increasing nurses’ knowledge in person-centred care | USA | Care Homes | 77 care home staff members | Quantitative. |
| King, Kelder, Phillips, McInerney, Doherty, Walls, Robinson & Vickers | Something for Everyone: MOOC Design for Informing Dementia Education and Research | 2013 | Conference: European Conference on E-Learning (ECEL) | To report on the development of a pilot Massive Open Online Courses (MOOC). The goal of the Understanding Dementia MOOC was to provide a foundation-level course that would increase evidence-based knowledge about dementia, internationally. | Australia | Higher Education | 128 participants: primarily from academia (nursing & midwifery) | Qualitative |
| King, O’Brien, Edelman & Fazio | Evaluation of the Person-Centered Care Essentials Program: Importance of Trainers in Achieving Targeted Outcomes | 2011 | Gerontology & Geriatrics Education | To describe a PCC training program and evaluation of program outcomes. In addition to examining impact of the training on participant knowledge and attitudes, we examine the effects of trainer confidence in site implementation ability on direct care workers’ (DCWs) knowledge of PCC. | USA | Care Homes and Domiciliary Care | 168 trainers, 1,782 care worker trainees | Quantitative |
| Kontos & Naglie | Expressions of Personhood in Alzheimer’s Disease: An Evaluation of Research-Based Theatre as a Pedagogical Tool | 2007 | Qualitative Health Research | To evaluate health care practitioners’ perceptions of the effectiveness of the research-based production in conveying the meaning and significance of bodily habits, gestures, and actions in the context of advanced stages of Alzheimer’s disease. | Canada | Hospital | 43 staff members: nurses, health care aides, occupational therapists, physiotherapists& recreational therapists | Mixed methods |
| Kontos, Mitchell, Mistry & Ballon | Using Drama to Improve Person-Centred Dementia Care | 2010 | International Journal of Older People Nursing | To discuss the qualitative evaluation of the effectiveness of the drama-based components of a 12-week drama-based educational intervention to introduce to dementia practitioners person-centred care that emphasises the notion of embodied selfhood (defined as non-verbal self-expression) | Canada | Care Homes | 24 nursing home practitioners | Qualitative |
| Kuske, Luck, Hanns, Matschinger, Angermeyer, Behrens & Riedel-Heller | Training in dementia care: a cluster-randomised controlled trial of a training programme for nursing home staff in Germany | 2009 | International Psychogeriatrics | To examine the effectiveness of a nursing home staff training programme designed to improve the interaction between residents with dementia and their caregivers. | Germany | Care Homes | 134 care staff and 321 residents | Quantitative |
| Lambert-Shute, Jarrott & Fruhauf | Service-Learning at Dementia Care Programs: An Orientation and Training Program | 2004 | Gerontology & Geriatrics Education | To describe a program designed to enhance the experiences of service-learners placed at a dementia care program through training, provision of structured activities, supervision, and follow-up | USA | Higher Education | 8 undergraduate students | Qualitative |
| Landreville, Dicaire, Verrault & Levesque | A training program for managing agitation of residents in long-term care facilities description and preliminary findings | 2005 | Journal of Gerontological Nursing | The purpose of this article is to describe a training program for managing agitation of long-term care residents and to report results of a pilot study. | France | Care Homes | 26 nursing staff, 19 residents | Quantitative |
| Lathren, Sloane, Hoyle, Zimmerman & Kaufe | Improving dementia diagnosis and management in primary care: a cohort study of the impact of a training and support program on physician competency, practice patterns, and community linkages | 2013 | BMC Geriatrics | To report the results of an interactive training and support program designed to educate primary care physicians and their staff on current evidence-based dementia clinical protocols and to link these practitioners to local dementia community resources. | USA | Primary Care | 29 Primary care physicians, 24 affiliated staff | Quantitative |
| Lea, Marlow, Bramble, Andrews, Crisp, Eccleston, Mason & Robinson | Learning Opportunities in a Residential Aged Care Facility: The Role of Supported Placements for First-Year Nursing Students | 2014 | Journal of Nursing Education | To examine whether novice nursing students can have a positive, quality clinical placement experience in the residential aged care environment, including the potential to learn about dementia, and to identify the factors that contribute to a positive experience | Australia | Higher Education | 30 First year nursing students | Mixed methods |
| Lea, Marlow, Bramble, Andrews, Eccleston, McInerney & Robinson | Improving student nurses’ aged care understandings through a supported placement | 2015 | International Nursing Review | To identify the potential for aged care placements to deliver benefits for second year nursing students when conducted within a supportive framework with debriefing and critical reflection opportunities. | Australia | Higher Education | 79 second year nursing students | Mixed methods |
| Lee, Weston & Hillier | Developing Memory Clinics in Primary Care: An Evidence-Based Interprofessional Program of Continuing Professional Development | 2013 | Journal Of Continuing Education in the Health Professions | The purpose of this article is to describe an innovative training program to build capacity within primary care settings for the assessment and management of dementia through the development of primary care–based memory clinics. | Canada | Primary Care | 124 Health professionals | Quantitative |
| Leone, Deudon, Bauchet, Laye, Bordone, Lee, Piano, Friedman, David, Delva, Brocker, Yesavage & Robert | Management of apathy in nursing homes using a teaching program for care staff: the STIM-EHPAD study | 2013 | International Journal of Geriatric Psychiatry | To evaluate the effectiveness of a nursing home (NH) staff education to manage apathy in older individuals with a diagnosis of dementia. | France | Care Homes | 230 residents and 563 caregivers | Mixed methods |
| Lerner, Resnick, Galik & Russ | Advanced Nursing Assistant Education Program | 2010 | Journal of Continuing Education in Nursing | To develop and test the effect of a full day advanced nursing assistant training course. | USA | Care Homes | 44 nursing assistants | Quantitative |
| Lintern, Woods & Phair | Before and after training: a case study of intervention. | 2000 | Journal of Dementia Care | To evaluate the extent to which staff training and development would improve resident well-being and QoL. | UK | Care Homes | 34 Care home assistants, 16 nurses, 44 residents | Quantitative |
| Litvin, Davis, Moran, Iverson, Zhao & Zapka | The Use of Clinical Decision-Support Tools to Facilitate Geriatric Education | 2012 | Journal of the American Geriatrics Society | To describe the development and use of clinical decision-support (CDS) tools to facilitate geriatric education and improve the care delivered to older adults in an academic internal medicine residency ambulatory care clinic. | USA | Hospital | 100 residents (medical students) and 17 faculty members supervising residents. | Quantitative |
| Liu, Pang & Lo | Development and implementation of an observational pain assessment protocol in a nursing home | 2012 | Journal of Clinical Nursing | To report the development and implementation of an Observational Pain Assessment Protocol in a Hong Kong nursing home and its impacts on pain management for cognitively impaired home residents (CIHRs). To report the opinions of the nursing home staff, including nurse assistants (NAs), nurses and a physiotherapist (PT), about the protocol. | Hong Kong | Care Homes | 11 nurses, 36 nursing assistants, 1 physiotherapist, 30 residents | Mixed methods |
| Long | Pain Management Education in Long-Term Care: It Can Make a Difference | 2013 | Pain Management Nursing | To identify whether staff education and training in pain management improve the knowledge, attitudes, and barriers related to pain management when providing care for older adults residing in long-term care. | USA | Care Homes | 91 staff including nurses, ancillary, other health professionals and direct care staff | Quantitative |
| MacDonald, Stodel & Casimiro | Online dementia care training forhealthcare teams in continuing and long-term care facilities: a viable solution for improving quality of care and quality of life for residents | 2006 | International Journal on e-learning | The purpose of this research was to design, develop, deliver and evaluate an online dementia care program aimed at enabling healthcare teams to deliver better service to residents with dementia in continuing and long-term care facilities. | Canada | Care Homes | 73 staff housekeeping staff to registered nurses. | Mixed methods |
| Mackenzie & Peragine | Measuring and enhancing self-efficacy among professional caregivers of individuals with dementia | 2003 | American Journal of Alzheimer’s Disease and Other Dementias | To describe the development and outcome of an intervention for long-term care nurses designed to decrease stress and burnout by enhancing their self-efficacy in managing challenging team, resident, and family situations. To present a self-efficacy inventory designed to measure the effectiveness of the intervention. | Canada | Care Homes | 41 nursing caregivers | Quantitative |
| Magai, Cohen & Gomberg | Impact of Training Dementia Caregivers in Sensitivity to Nonverbal Emotion Signals | 2002 | International Psychogeriatrics | To assess whether training caregivers in sensitivity to nonverbal communication could enhance mood and reduce symptoms in patients and improve psychological well-being in caregivers. | USA | Care Homes | 91 residents with dementia and 20 staff caregivers. | Quantitative |
| Mahendra, Freemant & Dionne | Teaching Future Providers about Dementia: The Impact of Service Learning | 2013 | Seminars in speech and language | To describe an innovative approach for providing speech-language pathology graduate students with exposure to long term care settings and clinical training in service delivery for persons with dementia. | USA | Higher Education | 45 Speech Language Pathology graduate students | Mixed methods |
| Markert, O’Neill & Bhatia | Using a Quasi-experimental Research Design to Assess Knowledge in Continuing Medical Education Programs | 2003 | The Journal of Continuing Education in the Health Professions | To assess knowledge acquired in continuing medical education (CME) programs | USA | Higher Education | 667 physicians, nurses, and other health professionals | Quantitative |
| McCabe, Bird, Davison, Mellor, MacPherson, Hallford & Seedy | An RCT to evaluate the utility of a clinical protocol for staff in the management of behavioral and psychological symptoms of dementia in residential aged-care settings | 2015 | Aging & Mental Health | To evaluate a training program to assist staff to manage behavioural and psychological symptoms of dementia in residential care | Australia | Care Homes | 187 residents, 204 staff members: personal care assistants, RGN/RNs, and others including lifestyle assistants and diversional therapists. | Quantitative |
| McCaffrey, Tappen, Lichtstein & Friedland | Interprofessional education in community-based Alzheimer’s disease diagnosis and treatment | 2013 | Journal of Interprofessional Care | To enhance medical and family nurse practitioner students’ basic competency in the detection and management of dementia. Use of an interprofessional approach to clinical education that would increase medical and nurse practitioner students’ understanding of the roles they and other professionals play in the delivery of care to individuals with Alzheimer’s disease and their families. | USA | Community | 80 second-year medical, 82 family nurse practitioner students | Quantitative |
| McCarthy | An evaluation of the effectiveness of Dementia Care Essentials in improving the quality of residential and community aged care | 2012 |  | To examine the effectiveness of Dementia Care Essentials training delivered at Certificate III and IV levels to aged care residential and community care staff, in improving the quality of aged care provided to people living with dementia. | Australia | Care Homes and Community | 59 care home staff and 47 residents | Quantitative |
| McCurry, LaFazia, Pike, Logsdon & Teri | Development and Evaluation of a Sleep Education Program for Older Adults with Dementia Living in Adult Family Homes | 2012 | American Journal of Geriatric Psychiatry | To investigate the feasibility of implementing a Sleep Education Program (SEP) for improving sleep in adult family home (AFH) residents with dementia, and the relative efficacy of SEP compared to usual care in a pilot randomised controlled trial. | USA | Care Homes | 37 staff, 47 residents. | Quantitative |
| McGilton, O’Brien-Pallas, Darlington, Evans, Wynn & Pringle | Effects of a Relationship-Enhancing Program of Care on Outcomes | 2003 | Journal of Nursing Scholarship | To examine the effects of a relationship-enhancing program of care (REPC) on resident and care provider outcomes. | Canada | Care Homes | 34 nursing staff, 40 residents | Quantitative |
| McPhail, Traynor, Wikstrom, Brown & Quinn | Improving outcomes for dementia care in acute aged care: impact of an education programme | 2009 | Dementia | To evaluate a dementia educational programme in a small local hospital offering, for the first time, an acute geriatric service and its impact on knowledge in a new specialist aged care clinical setting. | Australia | Hospital | 17 nurses 4 physiotherapists 2 pharmacists 2 social workers and 3 other. | Mixed methods |
| Mitchell, Dupuis, Jonas-Simpson, Whyte, Carson & Gillis | The Experience of Engaging With Research-Based Drama: Evaluation and Explication of Synergy and Transformation | 2011 | Qualitative Inquiry | To explore the influence of a research-based drama called I’m Still Here, in changing how persons understand, think about, and act with persons and families living with dementia. | Canada | All | 48 family members, 50 health care professionals, and 8 nursing students. | Qualitative |
| Monette, Champoux, Monette, Fournier, Wolfson, du Fort, Sourial, Le Cruguel & Gore | Effect of an interdisciplinary educational program on antipsychotic prescribing among nursing home residents with dementia | 2008 | International Journal of Geriatric Psychiatry | To assess the effect of an interdisciplinary educational program in reducing the use of antipsychotics in nursing home residents with dementia | Canada | Care Homes | 81 residents. 6 physicians, pharmacists, 74 nursing staff and 86 personal care assistants | Quantitative |
| Monette, J., Monette, M., Sourial, N., Vandal, A. C., Wolfson, C., Champoux, N., Fletcher, J., & Savoie, M. L. | Effect of an Interdisciplinary Educational Program on Antipsychotic Prescribing Among Residents With Dementia in Two Long-Term Care Centers | 2012 | Journal of Applied Gerontology | To assess the effect of our program on antipsychotic prescribing in two centres using an improved study design that included a pre-program control cohort within each centre, residents with dementia who were either users or nonusers of antipsychotics, and a post-program period to assess the long-term effect of the program. | Canada | Care Homes | 429 residents and 372 physicians, pharmacists, nursing staff and personal care attendants (PCAs) | Quantitative |
| Moorhouse & Hamilton | Not If, But When: Impact of a Driving and Dementia Awareness and Education Campaign for Primary Care Physicians | 2014 | Canadian Geriatrics Journal | To report on the impact of a provincial Web-based resource regarding driving cessation in dementia aimed towards primary care physicians (PCPs). | Canada | Primary Care | 134 primary care physicians | Quantitative |
| Narevic, Giles, Rajadhyax, Managuelod, Monis & Diamond | The effects of enhanced program review and staff training on the management of aggression among clients in a long-term neurobehavioral rehabilitation program | 2011 | Aging & Mental Health | To examine the effects of staff training, staff support groups and increased client behaviour monitoring on the rate of physical aggression toward peers and objects in an older neurobehavioral population treated in a long-term neurorehabilitation program | USA | Care Homes | 267 residents. Care home staff | Quantitative |
| National Museums Liverpool | An evaluation of National Museums Liverpool: Dementia Training Programme | 2012 | N/A | To describe and interpret in a final report the impacts for NML, partner organisations, the social and health care sector and those living and caring for people with dementia of the House of Memories training programme. | UK | All | 1200 health and social care workers | Mixed methods |
| Nayton, Fielding, Brooks, Graham & Beattie | Development of an Education Program to Improve Care of Patients With Dementia in an Acute Care Setting | 2014 | The Journal of Continuing Education in Nursing | To describe the development and delivery of a tailored education program to improve the quality of care of patients with dementia in a large, urban hospital in Australia. | Australia | Hospital | 45 nurses, 3 occupational therapists, 1 social worker | Quantitative |
| O'Shea, Devane, Cooney, Casey, Jordan, Hunter, Murphy, Newell, Connolly & Murphy | The impact of reminiscence on the quality of life of residents with dementia in long-stay care | 2014 | International Journal of Geriatric Psychiatry | To evaluate the effectiveness of a structured education-based reminiscence programme: the Dementia Education Programme Incorporating Reminiscence for Staff; for people with dementia residing in long-stay care settings in Ireland. | Ireland | Care Homes | Nursing and health care assistant staff, 304 residents | Quantitative |
| O'Sullivan & Hocking | Translating knowledge into practice: An exploratory study of dementia-specific training for community-based service providers | 2015 | Dementia | To develop, deliver, and evaluate dementia-specific training designed to inform service delivery by enhancing the knowledge of community-based service providers | New Zealand | Community | Management level staff and care staff from community (max. 48) | Qualitative |
| Palmer, Lach, McGillick, Murphy-White, Carroll, & Armstrong | The Dementia Friendly Hospital Initiative Education Program for Acute Care Nurses and Staff | 2014 | Journal of Continuing Education in Nursing | To examine changes in participants’ attitudes/ practices, confidence, knowledge and responses to the DFHI program, and to assure that outcomes from the prior testing in Phase 2 were maintained with further dissemination of the programme. | USA | Hospital | Nurses, therapists, social workers and chaplains. | Quantitative |
| Parks, Haines, Foreman, McKinstry & Maxwell | Evaluation of an Educational Program for Long-term Care Nursing Assistants | 2005 | Clinical Experience | To determine if an educational programme can improve knowledge and attitude among ancillary staff on end-of-life care issues in a long-term care facility. | USA | Care Homes | 32 ancillary staff | Quantitative |
| Passalacqua & Harwood | VIPS Communication Skills Training for Paraprofessional Dementia Caregivers: An Intervention to Increase Person-Centered Dementia Care | 2012 | Clinical Gerontologist | The aim of this study was to test the feasibility of a series of workshops built around VIPS, intended to increase the person-centred communication, beliefs, and attitudes among paraprofessional dementia caregivers in a long-term care facility. | USA | Care Homes | 26 staff caregivers | Quantitative |
| Pellfolk, Gustafson, Bucht & Karlsson | Effects of a Restraint Minimization Program on Staff Knowledge, Attitudes, and Practice: A Cluster Randomized Trial | 2010 | Journal of the American Geriatrics Society | To evaluate the effects of a restraint minimisation education program on staff knowledge and attitudes and use of physical restraints. | Sweden | Assisted Living | 346 Care staff and 353 residents | Quantitative |
| Pereles, Lockyer, Ryan, Davis, Spivak & Robinson. | The use of the opinion leader in continuing medical education | 2003 | Medical Teacher | Describes a process evaluation of an opinion leader project for a geriatric medical education program in dementia | USA | Hospital + Community | 13 physicians | Qualitative |
| Peterson, Berg-Weger, McGillick & Schwartz. | Basic Care I: The effect of dementia-specific training on certified nursing assistants and other staff | 2002 | American Journal of Alzheimer’s Disease and Other Dementias | To evaluate the impact of Basic Care I, a course that educates participants about the physiology of dementia, behavioral changes that accompany the disease, and how to cope with these behaviors. | USA | Care Homes | 72 Certified Nursing Assistants, Nurses, administrators, social workers, music therapists and porters. | Mixed methods |
| Resnick, Cayo, Galik & Pretzer-Aboff | Implementation of the 6-Week Educational Component in the Res-Care Intervention: Process and Outcomes | 2009 | The Journal of Continuing Education in Nursing | To evaluate the effects of a 6-week restorative care educational programme on knowledge for nursing assistants and assess satisfaction with the programme. | USA | Care Homes | 523 Nursing assistants. | Quantitative |
| Richardson, Kitchen & Livingstone | What staff know about elder abuse in dementia and the effect of training | 2004 | Dementia | To examine the knowledge and management of abuse of older people before and after two different educational interventions in staff who care for vulnerable older people. | UK | Community | 64 staff members | Quantitative |
| Richardson, Kitchen, & Livingston | The effect of education on knowledge and management of elder abuse: a randomized controlled trial | 2002 | Age and Ageing | To compare the effectiveness of attending an educational course to printed educational material in improving management of abuse of older people. To determine if positive attitude and low burnout scores are related to improvement. | UK | Community | 64 staff members: Nursing staff, care assistants, care managers and social workers. | Quantitative |
| Roberts & Gaspard | A palliative approach to care of residents with dementia | 2013 | Nursing Older People | To discuss the development and outcomes of an interprofessional pilot workshop for direct care providers held in a care home. | Canada | Care Homes | 53 care home and acute care staff | Mixed methods |
| Rodriguez, Marquett, Hinton, McBride, & Gallagher-Thompson | The Impact of Education on Care Practices: An Exploratory Study of Whether “Action Plans” Influence Health Professionals' Behavior | 2010 | International Psychogeriatrics | To ascertain the usefulness of “action plans” as a monitoring tool to evaluate concrete change over time, investigate factors that influence their successful implementation, and investigate factors that present challenges and barriers to their successful implementation. | USA | All | 366 Healthcare professionals and students | Mixed methods |
| Ruiz, Smith, van Zuilen, Williams, & Mintzer | The Educational Impact of a Computer-Based Training Tutorial on Dementia in Long Term Care for Licensed Practice Nursing Students | 2006 | Gerontology & Geriatrics Education | To evaluate the impact of training with the dementia computer-based training tutorial on licensed practice nursing students’ acquisition of knowledge of dementia care, attitudes toward dementia care, and self-efficacy ratings for specific dementia care skills, examine the relationship between self-efficacy ratings for skills and intention to apply learning to nursing practice, and assess learners’ perceptions of the dementia tutorial as a computer-based training tool. | USA | Higher Education | 38 graduating LPN students | Quantitative |
| Sackley, Rodriguez, Berg, Badger, Wright, Besemer, Reeuwijk & Wely | A phase II exploratory cluster randomized controlled trial of a group mobility training and staff education intervention to promote urinary continence in UK care homes | 2008 | Clinical Rehabilitation | To assess feasibility, acceptability and potential efficacy of group exercise and staff education intervention to promote continence in older people residing in care homes. To establish measures and information to inform a larger trial. | UK | Care Homes | 38 Care home staff and 33 residents | Quantitative |
| Schindel-Martin, Morden, Cetinski, Lasky, McDowell & Roberts | Teaching staff to respond effectively to cognitively impaired residents who display self-protective behaviors. | 2003 | American Journal of Alzheimer’s Disease and Other Dementias | To evaluate the effectiveness of a 7½ hour educational program designed to provide staff with the knowledge, skill, and confidence to manage physical self-protective behaviours of cognitively impaired long-term care residents. | Canada | Care Homes | 40 staff including professional and nonprofessional roles | Mixed methods |
| Schlaudecker, Lewis, Moore, Pallerla, Stecher, Wiebracht & Warshaw | Teaching Resident Physicians Chronic Disease Management: Simulating a 10- Year Longitudinal Clinical Experience With a Standardized Dementia Patient and Caregiver | 2013 | Journal of Graduate Medical Education | To describe the development, implementation, and evaluation of a chronic disease/geriatric medicine curriculum designed to teach Accreditation Council for Graduate Medical Education core competencies and geriatric medicine competencies to residents by using longitudinal encounters with a standardized dementia patient and her caregiver daughter. | USA | Higher Education | 134 medical students | Quantitative |
| Schrijnemaekers, van Rossum, Candel, Frederiks, Derix,Sielhorst & van den Brandt | Effects of emotion-oriented care on elderly people with cognitive impairment and behavioral problems | 2002 | International Journal of geriatric Psychiatry | To study the effects of emotion-oriented care on elderly people with moderate to severe cognitive impairment and behavioural problems and on professional caregivers in homes for the elderly. | Netherlands | Care Homes with a daycare unit | 151 residents, 64 care staff | Quantitative |
| Sidani, LeClerc & Streiner | Implementation of the abilities-focused approach to morning care of people with dementia by nursing staff | 2009 | International Journal of Older People Nursing | To determine the type and number of abilities-focused interventions delivered by nursing staff to residents with dementia during morning care. | Canada | Care Homes | 79 Registered Nursing staff, healthcare aides and personal support workers. | Quantitative |
| Sidani, Streiner, & LeClerc | Evaluating the effectiveness of the abilities-focused approach to morning care of people with dementia | 2012 | International Journal of Older People Nursing | To examine changes in resident outcomes before and after nursing staff’ implementation of the abilities-focused approach and the contribution of this approach to resident outcomes. | Canada | Care Homes | Nursing staff including registered nurses (RNs), registered practical nurses (RPNs) and personal support workers (PSWs), 65 residents | Quantitative |
| Skaalvik, Normann & Henriksen | Student experiences in learning person-centred care of patients with Alzheimer’s disease as perceived by nursing students and supervising nurses | 2010 | Journal of Clinical Nursing | To illuminate and discuss the experiences and perceptions of NSs and SNs regarding the students’ learning of person centred-care of patients with AD in a teaching nursing home (TNH) to make recommendations as to how student learning may be improved. | Norway | Care Homes | 13 Nursing students and supervisory staff | Qualitative |
| Skog, Negussie & Grafström | Learning dementia care in three contexts: practical training in day-care, group dwelling and nursing home | 2000 | Journal of Advanced Nursing | To illuminate how trainees utilise their practical training to learn about dementia care. | Sweden | Care Homes, Community | 18 licensed practical nurses (LPNs) | Qualitative |
| Sloane | Effect of a Person-Centered Mouth Care Intervention on Care Processes and Outcomes in Three Nursing Homes | 2013 | The American Geriatrics Society | To develop and test a person-centred, evidence-based mouth care program in nursing homes. | USA | Care Homes | 6 Nursing Assistants, 97 residents | Quantitative |
| Smythe, Jenkins, Harries, Atkins, Miller, Wright, Wheeler, Dee, Bentham & Oyebode | Evaluation of dementia training for staff in acute hospital settings | 2014 | Nursing Older People | To develop, pilot and evaluate a brief psychosocial training intervention (BPTI) for staff working with people with dementia in an acute hospital setting. | UK | Hospital | 81 members of ward staff | Mixed methods |
| Söderlund, Norberg & Hansebo | Validation method training: nurses’ experiences and ratings of work climate | 2014 | International Journal of Older People Nursing | To explore nurses’ experiences of a 1-year validation method (VM) training programme conducted in a nursing home for residents with dementia and to describe ratings of work climate before and after the programme. | Sweden | Care Homes | 12 nurses | Mixed methods |
| Speziale, Black, Coatsworth-Puspoky, Ross, & O’Regan. | Moving Forward: Evaluating a Curriculum for Managing Responsive Behaviors in a Geriatric Psychiatry Inpatient Population | 2009 | The Gerontologist | To assess the impact of the Gentle Persuasive Approaches (GPA) curriculum on staff knowledge and competency and also on patient risk events and occupational health incidents. | Canada | Hospital | 99 staff: Domestic Services, Psychology, Social Work, Spiritual Care, Occupational Therapy, Therapeutic Recreation, Nursing, and Clerical. | Quantitative |
| Stevens-Roseman & Leung | Enhancing Attitudes, Knowledge, and Skills of Paraprofessional Service Providers in Elder Care Settings | 2004 | Gerontology & Geriatrics Education | To implement and evaluate a training program to enhance the gerontological attitudes, knowledge and skills of paraprofessional service providers working in elder care settings. | USA | Community | 74 staff members: nurse aides, certified nursing assistants, activity directors, receptionists, homemakers, and family respite aides. | Quantitative |
| Struck, Bernard & Teasdale | Effect of a Mandatory Geriatric Medicine Clerkship on Third-Year Students | 2005 | Journal of the American Geriatrics Society | To assess the impact of a mandatory geriatric medicine clerkship on third-year students | USA | Higher Education | 135 medical students | Mixed methods |
| Surr, Smith, Crossland & Robins | Impact of a person-centred dementia care training programme on hospital staff attitudes, role efficacy and perceptions of caring for people with dementia: A repeated measures study | 2015 | International Journal of Nursing Studies | To evaluate the efficacy of a specialist training programme for acute hospital staff regarding improving attitudes, satisfaction and feelings of caring efficacy, in provision of care to people with dementia. | UK | Hospital | 40 acute hospital staff working in clinical roles (90% nurses) | Quantitative |
| Szymczynska & Innes | Evaluation of a dementia training workshop for health and social care staff in rural Scotland | 2011 | Rural and Remote Health | To explore the learning needs and outcomes of training participants delivering services to people with dementia in a rural area. | UK | All | 18 Health and social staff | Qualitative |
| Tannazzo, Breuer, Williams & Andreoli | A Dementia Training Program to Benefit Certified Nurse Assistant Satisfaction and Nursing Home Resident Outcomes | 2008 | Alzheimer's Care Today | To disseminate best practice in dementia behaviour management on-site in 4 New York City area nursing homes, in the hopes of increasing CNA satisfaction through knowledge acquisition. | USA | Care Homes | 301 Certified nursing assistants, 534 residents | Quantitative |
| Teresi, Ramirez, Silver, Boratgis, Kong, Eimicke, Pillemer & Lachs | A staff intervention targeting resident-to-resident elder mistreatment (R-REM) in long-term care increased staff knowledge, recognition and reporting: Results from a cluster randomized trial | 2013 | International Journal of Nursing Studies | To evaluate the impact of a newly developed R-REM training intervention for nursing staff on knowledge, recognition and reporting of R-REM. | USA | Care Homes | 658 (approx.) Certified nursing assistants and nursing staff, 1405 residents | Quantitative |
| Teri, Huda & Gibbons | Improving Dementia Care in Assisted Living Residences: Addressing Staff Reactions to Training | 2009 | Geriatric Nursing | To investigate whether the STAR—Staff Training in Assisted-living Residences training would be feasible and relevant in diverse assisted living residences (ALRs) and to determine whether relatively novice trainers could be taught to train staff consistently and effectively and whether outcomes obtained by these trainers at these diverse ALR sites would be comparable to the positive outcomes obtained in earlier trials. | USA | Assisted Living | 44 Certified Nursing Assistants | Mixed methods |
| Teri, McCurry, Logsdon & Gibbons | Training Community Consultants to Help Family Members Improve Dementia Care: A Randomized Controlled Trial | 2005 | The Gerontologist | To assess whether community consultants could be trained to teach family caregivers a systematic behavioural approach for reducing mood and behaviour problems in persons with Alzheimer’s disease. | USA | Community | 6 Community health care professionals, 95 individuals with dementia | Quantitative |
| Teri, Huda, Gibbons, Young & van Leynseele | STAR: A Dementia-Specific Training Program for Staff in Assisted Living Residences | 2005 | The Gerontologist | To describe, and provide data on an innovative, comprehensive, dementia-specific training program designed to teach direct care staff in assisted living residences to improve care and reduce problems in residents with dementia. | USA | Assisted Living | 114 Direct care staff, 120 residents with dementia | Quantitative |
| Testad, Aasland & Aasland | The effect of staff training on the use of restraint in dementia: a single-blind randomised controlled trial | 2005 | International Journal of Geriatric Psychiatry | To reduce behavioural symptoms and the use of restraint in residents with dementia using a staff training program as intervention. | Norway | Care Homes | 151 residents, care staff | Quantitative |
| Testad, Mekki, Førland, Øye, Tveit Jacobsen & Kirkevold | Modelling and evaluating evidence-based continuing education program in nursing home dementia care (MEDCED)—training of care home staff to reduce use of restraint in care home residents with dementia. A cluster randomized controlled trial | 2015 | International Journal of Geriatric Psychiatry | To evaluate the effectiveness of a tailored 7-month training intervention “Trust Before Restraint,” in reducing use of restraint, agitation, and antipsychotic medications in care home residents with dementia. | Norway | Care Homes | All staff working at the care home; 274 residents with dementia | Quantitative |
| Thompson & Devenney | Training in dementia for primary care professionals: the role ofthe Admiral Nurse | 2007 | Primary Health Care | To describe a project to educate primary care professionals in addressing the needs of people with dementia and their families and carers | UK | Primary Care | All primary care unit staff | Qualitative |
| van der Kooij, Dröes, de Lange, Ettema, Cools & van Tilburg. | The implementation of integrated emotion-oriented care: Did it actually change the attitude, skills and time spent of trained caregivers? | 2013 | Dementia | To assess whether the trained caregivers actually applied the knowledge and techniques of IEOC during their daily work, and whether offering IEOC is more time consuming for care personnel than usual care. | Netherlands | Care Homes | 124 professional carers | Mixed methods |
| van Zuilen, Mintzer, Milanez, Kaiser, Rodriguez, Paniagua, Ruiz & Roos | A Competency-Based Medical Student Curriculum Targeting Key Geriatric Syndromes | 2008 | Gerontology & Geriatrics Education | This paper describes the design, development, implementation, and evaluation of a competency-based undergraduate medical education (UME) curriculum, reviews data-driven curriculum quality improvement efforts, and discusses the challenges to translating student competency into routine practice. | USA | Higher Education | 793 students | Quantitative |
| Vanlaere, Timmermann, Stevens & Gastmans | An explorative study of experiences of healthcare providers posing as simulated care receivers in a ‘care-ethical’ lab | 2012 | Nursing Ethics | To gain insight into the impact of empathy sessions on the empathic abilities of care providers who underwent an empathy session in the sTimul care-ethics lab as simulated patients. | Belgium | Higher Education | 15 care providers: 7 Registered Nurses, 4 cleaning or logistics assistants, 3 healthcare assistants, 1 kinesistherapist | Qualitative |
| Velzke | Evaluation of a dementia care learning programme | 2014 | Nursing Older People | This article reports on an evaluation of the Dementia Services Development Centre’s Best Practice in Dementia Care Learning Programme for hospitals, care homes including day centres and domiciliary care settings. | UK | All | 100 service managers | Mixed methods |
| Vida, Monette, Wilchesky, Monette, Friedman, Nguyen, Dastoor, Cristache, Sourial, Tremblay & Gore | A long-term care center interdisciplinary education program for antipsychotic use in dementia: program update five years later | 2012 | International Psychogeriatrics | To assess rates of AP use five years after our first intervention to determine the long-term impact; and to implement an updated AP reduction educational intervention program at the same centre five years later in order to determine whether AP use could be further reduced. | Canada | Care Homes | Care home staff, 46 residents | Quantitative |
| Vollmar, Mayer, Ostermann, Butzlaff, Sandars, Wilm & Rieger | Knowledge transfer for the management of dementia: a cluster-randomised trial of blended learning in general practice | 2010 | Implementation Science | To compare knowledge acquisition about dementia management between a blended learning approach using online modules in addition to quality circles (QCs) and QCs alone. | Germany | Primary Care | 166 General Practitioners | Quantitative |
| Warshaw, Modawal, Kues, Moore, Margolin, Sehgal, Mueller & Cluxton | Community Physician Education in Geriatrics: Applying the Assessing Care of Vulnerable Elders Model with a Multisite Primary Care Group | 2010 | Journal of the American Geriatrics Society | To increase Alliance Primary Care (APC) physicians’ clinical skills and assist them in implementing new office and system strategies to improve the quality of their care of older patients. | USA | Primary Care | 60 Primary care physicians | Mixed methods |
| Wesson & Chapman | A dementia education scheme | 2010 | Nursing Older People | To provide education for all levels of staff throughout the county’s health system and community, teaching about conditions that involve communication, memory or reasoning difficulties and providing simple, practical strategies for working with patients with such difficulties. | UK | Hospital | 300 hospital staff | Qualitative |
| Westmoreland, Counsell, Tu, Wu & Litzelman | Web-Based Training in Geriatrics for Medical Residents: A Randomized Controlled Trial Using Standardized Patients to Assess Outcomes | 2010 | Journal of the American Geriatrics Society | To compare knowledge of residents after Web-based with their knowledge after paper-based instruction covering geriatrics content and to compare residents’ clinical application of their Web-based knowledge with that of their paper-based knowledge using unannounced SPs and ASPs. | USA | Higher Education | 96 Postgraduate medical students | Quantitative |
| Wilcock, Iliffe, Griffin, Jain, Thuné-Boyle, Lefford & Rapp | Tailored educational intervention for primary care to improve the management of dementia: the EVIDEM-ED cluster randomized controlled trial | 2013 | Trials | To test the effect of a workplace-based tailored educational intervention developed for general practice on the clinical management of people with dementia. | UK | Primary Care | General Practitioners, 1072 people with dementia | Quantitative |
| Williams | Improving Outcomes of Nursing Home Interactions | 2006 | Research in Nursing & Health | To evaluate the effects of a three-session program based on the communication enhancement model by comparing audio recordings of staff-resident communication collected before and immediately, and 2 months after the intervention. | USA | Care Homes | 38 Direct care staff, 60 residents | Quantitative |
| Zimmerman, Mitchell, Reed, Preisser, Fletcher, Beeber, Reed, Gould, Hughes, McConnell, Corazzini, Lekan & Sloane | Outcomes of a Dementia Care Training Program for Staff in Nursing Homes and Residential Care/Assisted Living Settings | 2010 | Alzheimer's Care Today | To report the results of a nested cohort group-randomized trial of 6 training sessions of Foundations of Dementia Care, a national training curriculum for nursing home and residential care/assisted living staff. | USA | Care Homes + Assisted Living | Minimum 600 care staff | Quantitative |
| Zwijsen, Gerritsen, Eefsting, Smalbrugge, Hertogh & Pot | Coming to grips with challenging behaviour: A cluster randomised controlled trial on the effects of a new care programme for challenging behaviour on burnout, job satisfaction and job demands of care staff on dementia special care units | 2015 | International Journal of Nursing Studies | To determine the effects of a care programme for the challenging behaviour of nursing home residents with dementia on the burnout, job satisfaction and job demands of care staff. | Netherlands | Care Homes | 380 Staff members | Quantitative |
