# Supplementary material for: Effective Dementia Education and Training for the Health and Social Care Workforce: A Systematic Review of the Literature
Source: Rev Educ Res. 2017 Jul 31;87(5):966–1002. doi: 10.3102/0034654317723305 (PMC5613811; doi:10.3102/0034654317723305)
Supplement: Supplementary material [file Table_S2_Included_studies_quality_and_reported_outcomes_online_supp.docx]

Table S2:

*Included studies quality rating and reported outcomes*

| **Authors** | **Title** | **Year** | **Training features** | **Kirkpatrick outcomes reported** | | | | **Qual score** | **Qual cat** |
| --- | --- | --- | --- | --- | --- | --- | --- | --- | --- |
|  |  |  |  | **Reaction** | **Learning** | **Behaviour** | **Results** |  |  |
| Alnes, Kirkevold & Skovdahl | Insights gained through Marte Meo counselling: experiences of nurses in dementia specific care units. | 2011 | Marte Meo (one’s own strength) Counselling. Videotaped interactions of care situations. |  | + knowledge |  |  | 11 | 3 |
| Alnes, Kirkevold & Skovdahl | The influence of the learning climate on learning outcomes from Marte Meo counselling in dementia care | 2013 | Marte Meo (one’s own strength) Counselling. Videotaped interactions of care situations. Reflective discussions. | + overall  - Questioned relevance, hard to find time to attend | + knowledge | + general practice changes |  | 10 | 2 |
| Amos, Bush, Byszewski, Dalziel, Graham, Guzman, Hunt, Man-Son-Hing & Marshall | A continuing medical education initiative for canadian primary care physicians: the driving and dementia toolkit | 2003 | The driving and dementia toolkit: A toolkit (booklet) of topical background info, local resources, safety screening questions and FAQs for self-directed use. | + satisfaction | + knowledge | + driving assessment |  | 11 | 3 |
| Arcand, Monette, Monette, Sourial, Fournier, Gore & Bergman | Educating Nursing Home Staff About the Progression of Dementia and the Comfort Care Option: Impact on Family Satisfaction with End-of-Life Care | 2009 | Didactic consciousness-raising and educational sessions. Classroom based plus booklet on comfort care. |  |  |  | - communication with families | 10 | 2 |
| Asthill | Staff training and challenging behaviour in a day hospital | 2004 | Didactic, classroom based. Four modules presented as informal, interactive and discussion-based workshops on issues concerning a person’s behaviour in care. | + well received |  |  | + behavior of person with dementia | 7 | 2 |
| Barbosa, Nolan, Sousa & Figueiredo | Supporting direct care workers in dementia care: effects of a psychoeducational intervention | 2015 | Evidence-based psychoeducational intervention (educative and supportive components). Multi-approach: Meditation, yoga, stretching exercises. Conflict management, emotional support, life tools coaching, problem solving techniques. |  | + knowledge |  | + staff emotional exhaustion  - staff stress and job satisfaction | 13 | 3 |
| Beer, Horner, Flicker, Scherer, Lautenschlager, Bretland, Flett, Schaper & Almeida | A Cluster-Randomised Trial of Staff Education to Improve the Quality of Life of People with Dementia Living in Residential Care: The Direct Study | 2011 | Didactic, classroom based and self-directed + Champions. 30-minute sessions. Content included communication, personal care and activities, positive values, behaviours of concern, pain management, the ‘‘3 Ds’’ (dementia, depression and delirium), and effective working between GPs and Residential Aged Care Facility. | + met training needs |  | + GP training reduced restraint  + pain assessments with care home staff training  - pain assessments with GP training  + case conference frequency in care home staff training  - comprehensive medical assessments with GP training | - resident quality of life  + GP training reduced observed resident pain | 11 | 3 |
| Beer, Lowry, Horner, Almeida, Scherer, Lautenschlager, Bretland, Flett, Schaper & Flicker | Development and evaluation of an educational intervention for general practitioners and staff caring for people with dementia living in residential facilities | 2011 | 27 lessons on aspects of dementia care. Identifying and supporting Dementia Champions. GP education program consisted of five “lessons”, final held as a reflective session to consolidate principles. Active engagement with facility managers. Supporting Materials including posters and resource packs. | + met training needs  + use of video clips  + use of scenarios   - Sessions too short |  | + person-centred care and delivery of personal care |  | 9 | 2 |
| Beer, Hutchinson & Skala-Cordes | Communicating With Patients Who Have Advanced Dementia: Training Nurse Aide Students | 2012 | Didactic, classroom based. 45-minute training on enhanced communication techniques with people with advanced dementia. Presenters demonstrated through video- and audio-taped examples. |  | + knowledge |  |  | 10 | 2 |
| Beville | Virtual Dementia Tour Helps Sensitize Health Care Providers | 2002 | Simulation: A preparation period followed simulated scenario. Participants required to wear items of clothing whilst completing simple tasks to induce discomfort, disorientation and impair motor skills, intended to mimic the experience of dementia. | +/- intense emotional reactions, expressed ‘bizarre’ and ‘inappropriate’ behaviours | +/- attitudes (excluded from analysis) |  |  | 4 | 1 |
| Bluethmann | Enhancing Quality Through Staff Training | 2008 | Classroom based. Evidence-based practices, Flexible design, experiential learning, teamwork emphasis, person-centred care focus. 12 1-hour sessions in 5 modules: About Dementia, Learning to Lead, Enhancing Mealtime, Reducing Pain, and Making Connections. Handouts and summary cards. | + met learning needs  Preference for topics relevant to role and with practical applications | + knowledge  + confidence  - attitudes |  |  | 6 | 2 |
| Bourgeois, Dijkstra, Burgio & Allen | Communication Skills Training for Nursing Aides of Residents with Dementia: The Impact of Measuring Performance | 2004 | Classroom + in-service mentoring with informative materials. Combined communication enhancing intervention (with memory aids) and one-on-one staff-management program on the quantity and quality of communicative interactions. |  |  | + reduce anti-psychotic prescribing |  | 12 | 3 |
| Brooker, Latham, Evans, Jacobson, Perry, Bray, Ballard, Fossey, & Pickett | FITS into practice: translating research into practice in reducing the use of anti-psychotic medication for people with dementia living in care homes | 2015 | Champion, cascade model: 10-day, standardised face-to-face education programme. Focused on critical reflection, peer support and continued implementation and improvement. Included strengths-based care planning, life story work, supportive environments and meaningful activity | + positive response to programme  - stressful if unable to apply learning in practice | + knowledge  + confidence  + attitudes | + reduced anti-psychotic prescribing |  | 8 | 2 |
| Broughton, Smith, Baker, Angwin, Pachana, Copland, Humphreys, Gallois, Byrne & Chenery | Evaluation of a caregiver education program to support memory and communication in dementia: A controlled pretest–posttest study with nursing home staff | 2011 | 50 min DVD containing info on difficulties observed in dementia, and straightforward, evidence-based strategies (entitled RECAPS and MESSAGE) to assist caregivers in maximising everyday memory and communication. Includes summary booklet and posters + lanyards on the strategies. | + useful, applicable  + content clear, concise  + liked DVD examples  - less applicable to people with more advanced dementia | + knowledge maintained at 3-month follow-up |  |  | 12 | 3 |
| Brown Wilson, Swarbrick, Pilling, & Keady | The senses in practice: enhancing the quality of care for residents with dementia in care homes | 2013 | Classroom based workshops. Uses a framework based on the Senses in Practice study. Participants encouraged to adopt problem-solving approaches to consider how they might enhance the experience of the person with dementia by creating each of the Senses. |  | + confidence | + initiation of meaningful conversations |  | 6 | 2 |
| Burgess & Page | Educating nursing staff involved in the provision of dementia care | 2003 | Classroom and in-service mentoring. Traditional didactic approach. Working on the wards delivering situation-based education and role modelling. |  | + knowledge  + confidence |  | - carer satisfaction | 4 | 1 |
| Burgio, Allen-Burge, Roth, Bourgeois, Dijkstra, Gerstle, Jackson & Bankster | Come Talk With Me: Improving Communication Between Nursing Assistants and Nursing Home Residents During Care Routines | 2001 | Classroom + in-service. All nursing staff trained in the use of memory books and general communication skills. Residents received a personalized 12-page memory book containing biographical, orientation, and daily schedule information. |  |  | + staff communication skills | - resident rate of interactions | 11 | 3 |
| Burgio, Stevens, Burgio, Roth, Paul & Gerstle | Teaching and Maintaining Behavior Management Skills in the Nursing Home | 2002 | Classroom/In-service + feedback. Nursing staff received behavior management skills training through in-service classes and hands-on training over 4 weeks. |  | + knowledge | +/- staff communication skills  - staff physical assistance approaches | - resident independence  - resident agitation | 12 | 3 |
| Cameron, Horst, Lawhorne & Lichtenberg | Evaluation of academic detailing for primary care physician dementia education | 2010 | Academic detailing - face-to-face visits with individuals by a peer. A series of 15-minute educational sessions delivered to physicians and their staff by a trained team. Physicians received a packet of materials including triggers for identifying potential dementia cases. | + well designed  - substantial variation in delivery by network of trainers | + knowledge | + referral rates |  | 6 | 1 |
| Cartwright, Franklin, Forman & Freegard | Promoting collaborative dementia care via online interprofessional collaboration | 2013 | Classroom + experiential learning. Clinical scenario and structured activities to foster interactive problem solving and shared clinical reasoning each aligned with a group investigation. Online case study run over 4 weeks with new info introduced each week. |  | + knowledge |  |  | 9 | 2 |
| Chang & Lin | Effects of a feeding skills training programme on nursing assistants and dementia patients | 2005 | Classroom and in-service. Class content: overview of dementia, etiology and behaviours of feeding among dementia patients and protocol for feeding dementia patients. Written manual of programme. Classroom training followed immediately by hands-on training. |  | + knowledge  + attitude | + approaches to supporting resident to eat | + took more time over resident meals  - resident food intake | 11 | 3 |
| Chang, Wykle & Madigan | The Effect of A Feeding Skills Training Program for Nursing Assistants Who Feed Dementia Patients in Taiwanese Nursing Homes | 2006 | Classroom and in-service (see above for content). Nursing assistants were taught using the Hellen approach with a series of activities to promote feeding behaviors. The class was followed immediately by a 1-hour hands-on training. |  | + knowledge  - attitude | + approaches to supporting resident to eat | + took more time over resident meals |  |  |
| Chenoweth, King, Jeon, Brodaty, Stein-Parbury, Norman, Haas & Luscombe | Caring for Aged Dementia Care Resident Study (CADRES) of person-centred care, dementia-care mapping, and usual care in dementia: a cluster-randomised trial | 2009 | Classroom based and practical implementation; Person-centred care approaches and Dementia Care Mapping. Two trained mappers at each site and mappers encouraged changes to be implemented in residents’ care plans. |  |  | - staff communication  - anti-psychotic prescribing | + reduced agitation  + reduced falls for DCM training  - other resident behaviours  - resident quality of life | 12 | 2 |
| Clare, Whitaker, Woods, Quinn, Jelley, Hoare, Woods, Downs & Wilson | AwareCare: a pilot randomized controlled trial of an awareness-based staff training intervention to improve quality of life for residents with severe dementia in long-term care settings. | 2013 | Classroom + practice project. Care staff received training and supervision, and observed selected residents according to a predefined schedule using a structured observational measure. Care staff in each home participated in two 90- minute training sessions led by an accredited trainer. | + staff felt benefitted from training, liked opportunity to spend time observing individual residents | + knowledge | + person-centred care | + resident quality of life | 11 | 3 |
| Coogle, Head & Parham | Person-centred care and the workforce crisis: a statewide professional development initiative. | 2004 | Train-the-trainer model. Curriculum: Person-centred care - Skill building for caregivers of people with dementia. 12-hour basic multidisciplinary training programme comprised of 6 modules. Student manuals also used. | + largely positive learner reactions to all aspects of training | + knowledge |  |  | 5 | 1 |
| Coogle, Head & Parham | The long-term care workforce crisis: Dementia-care training influences on job satisfaction and career commitment | 2006 | Train-the-trainer model (see above for description). |  |  |  | + job satisfaction  - staff commitment | 6 | 2 |
| Cooke, Moyle, Venturato, Walters & Kinnane | Evaluation of an education intervention to implement a capability model of dementia care | 2014 | Classroom + in-service. Two registered nurses provided face-to-face sessions over first two months and on-site mentorship for 6 months. The research team provided extensive education for these two facilitators consisting of workshops on dementia, mentorship, leadership and change, reflective journaling. | + training well designed, understandable and relevant  + opportunities for discussion |  |  |  | 8 | 2 |
| Corwin, Owen & Perry | Student service learning and dementia: bridging classroom and clinical experiences | 2008 | In-service vs in-service + classroom. Nursing students (control) instructed to visit CH residents weekly up to 45 hours and interact with them to the best of their abilities. They received a 3-hour lecture on communication with older adults with dementia. Speech-language pathology students (experimental group) as above but created a personalised “connection kit” for each resident, based on cognitive linguistic communication. | + more responsible for learning,  - felt needed more classroom time on dementia, service learning not directly linked to building clinical skills | + knowledge |  |  | 7 | 2 |
| Dalsgaard, Kallerup & Rosendal | Outreach visits to improve dementia care in general practice: a qualitative study | 2007 | Classroom + written resource. GP facilitators and gerontopsychiatrists devised a leaflet with main messages about dementia. Faciitator visited GP once. | + general positive response to training | + knowledge  + confidence |  |  | 9 | 2 |
| Davies, Lambert, Turner, Jenkins, Aston & Rolfe | Making a difference: using action research to explore our educational practice | 2014 | Classroom based, not described in further detail. | + trainer qualities including safe learning environment where learners can ask questions  - wanted more experiential learning and video clips | + knowledge |  |  | 10 | 2 |
| Davison, McCabe, Visser, Hudgson, Buchanan & George | Controlled trial of dementia training with a peer support group for aged care staff | 2007 | Classroom + experiential. Eight sessions of 60-90 minutes. Combination of didactic and experiential learning. Focused on skills to use in caring for residents with dementia-related behaviours, delivered by experienced mental health clinicians. Included a peer support program. |  | + confidence | + general practice improvement | - resident behaviours  - staff emotional exhaustion, depresonalisation or satisfaction | 10 | 2 |
| Deudon, Maubourguet, Gervais, Leone, Brocker, Carcaillon, Riff, Lavallar & Robert | Non- pharmacological management of behavioural symptoms in nursing homes | 2009 | Classroom + in-service. 24 hours total over 8-weeks Initial 90-min teaching session - dementia, symptoms and use of 'how-to' instruction cards (for staff). Trainers provided constructive feedback on how staff dealt with BPSD and emphasised use of instruction cards. |  |  |  | + reduced resident agitation | 11 | 3 |
| Downs, Turner, Bryans, Wilcock, Keady, Levin, O'Carroll, Howie & Iliffe | Effectiveness of educational interventions in improving detection and management of dementia in primary care: cluster randomised controlled study | 2006 | Individual DVD vs decision support vs classroom. Electronic tutorial using a cd-rom, Decision support software, practice-based workshops with a standard curriculum designed by an expert panel. |  |  | + detection of dementia  - concordance with guidelines |  | 12 | 3 |
| Edvardsson, Sandman & Borell | Implementing national guidelines for person-centered care of people with dementia in residential aged care: effects on perceived person-centeredness, staff strain, and stress of conscience | 2014 | Classroom, action learning. Two introductory day-long seminars. The first was devoted to presenting the Swedish national guidelines for dementia care. The second was a workshop where staff were asked to analyse their unit’s strengths, weaknesses, and threats in relation to the national guidelines, and present a topic for their unit-based knowledge generation process. |  |  | + person-centred care | + reduced staff stress  - staff job strain | 12 | 3 |
| Edwards, Voss & Iliffe | The development and evaluation of an educational intervention for primary care promoting person-centred responses to dementia | 2013 | Training delivered by the researcher over a lunch time meeting. Each participant given a printed and bound handbook and case examples and the researcher talked through the PowerPoint presentation using the guidelines set out in the training manual. 1 hr max session. |  | + knowledge  + confidence |  |  | 6 | 2 |
| Elliott & Adams | Using a practice development project to improve standards of care for people with dementia | 2012 | Classroom based. Five core sessions, each lasting three hours: What is dementia? Person-centred care; Communication; Activities; Managing complex behaviours. Formal training sessions and ‘working alongside’ modelling support available to staff. | + staff appreciated training | + attitudes |  |  | 3 | 1 |
| Ellis | Carer-driven dementia education for professionals | 2008 | Group DVD. Training was delivered in 4 sessions, of approx.. 75 minutes, with the carers featured in the DVD present. Each session included 4 video accounts, followed by questions, comments and discussion. | + positive feedback, seen as valuable | + knowledge |  |  | 1 | 1 |
| Elvish, Burrow, Cawley, Harney, Graham, Pilling, Gregory, Roach, Fossey & Keady | ‘Getting to Know Me’: the development and evaluation of a training programme for enhancing skills in the care of people with dementia in general hospital settings | 2014 | Classroom based. Introduction to dementia, seeing the whole person, Communication skills, impact of the hospital environment, knowing the person, person centred understanding of challenging behaviour. Designed to be delivered flexibly; delivered in four 45-90 minute sessions. |  | +/- statistically but not clinically significant improvements in knowledge and confidence |  |  | 13 | 3 |
| Emerson Lombardo, Wu, Hohnstein & Chang | Chinese Dementia Specialist Education Program: Training Chinese American Health Care Professionals as Dementia Experts | 2002 | Classroom Based. 8 classes over a 2.5 month period. Lectures followed by a brief discussion. The final 2 1/2 classes designed to encourage staff to support and take ownership of any future new dementia outreach efforts and services initiated by agencies | +/- wanted more case studies, tests and quizzes and how to administer assessments | + knowledge  + confidence | + general practice improvements |  | 5 | 1 |
| Engelman, Altus, Mosier and Mathews | Brief training to promote the use of less intrusive prompts by nursing assistants in a dementia care unit | 2003 | Classroom + in-service. 30-min training workshop to learn how to implement SLP approach during morning dressing routines. Researchers role-played the SLP procedure while the CNA watched, then provided feedback to the CNA after three additional roleplaying interactions and again in real life situations. |  |  | + staff communication  + personal care delivery |  | 3 | 1 |
| Ervin & Koschel | Dementia care mapping as a tool for person centred care | 2012 | Classroom. Training over three consecutive days by staff from the University of Bradford. DCM + feedback delivered locally by managers |  | + attitudes | + reduced anti-psychotic prescribing |  | 5 | 1 |
| Featherstone, James, Powell, Milne & Maddison | A controlled evaluation of a training course for staff who work with people with dementia | 2004 | Training combined components of cognitive behaviour therapy, experiential learning and didactic sessions to convey facts about dementia. Staff were encouraged to develop strategies acceptable to both the residents and the staff. Teaching employed feedback (both giving and eliciting), good pacing, empathy, humour and modelling. |  | + knowledge  + attitudes |  | - staff coping | 8 | 2 |
| Figueiredo, Barbosa, Cruz, Marques & Sousa | Empowering Staff in Dementia Long-Term Care: Towards a More Supportive Approach to Interventions | 2013 | Classroom + psychoeducational. Staff provided with specialised knowledge regarding dementia and providing care. Supportive component aimed to help staff to manage the emotional demands of working in dementia care. Case examples based on staff past experiences, group discussions, home-work exercises, role-playing, and brainstorming. | + having facilitator who listens as well as teaches  + informative and applicable, clear and well structured | + knowledge  + confidence  + attitudes |  | - amount of resident communication | 7 | 2 |
| Finnema, de Lange, Droes, Ribbe & van Tilburg | The quality of nursing home care: do the opinions of family members change after implementation of emotion-oriented care? | 2001 | Classroom + supervision. Addressed aspects of approaching one's work methodically, and drawing up individual care plans for each resident. One staff member per unit asked to become a ward adviser encouraging staff to work according to the principles of the Model-Care plan. Advisers received a minimum of three half days’ supervision in the workplace. |  | + knowledge |  | - carer satisfaction | 13 | 3 |
| Finnema, Droes, Erttema, Ooms, Ader, Ribbe & van Tilburg | The effect of integrated emotion-oriented care versus usual care on elderly persons with dementia in the nursing home and on nursing assistants: a randomized clinical trial | 2005 | Model-Care plan training course (see above). |  | - confidence |  | + reduced staff stress reactions  - staff perceived work-related stress  +/- reduced resident anxiety and improved quality of life in those with less severe dementia, no change for those with more severe dementia | 14 | 3 |
| Fossey, Ballard, Juszczak, James, Alder, Jacoby & Howard | Effect of enhanced psychosocial care on antipsychotic use in nursing home residents with severe dementia: cluster randomised trial | 2006 | Classroom + in-service + supervision, practice work. Package involved a systemic consultation approach. This tackled “whole home” issues, such as environmental, care practice, and attitudinal factors. Clinicians supported the use of activities through didactic training, skills modelling, and supervision of groups and individual staff. |  |  | + reduced anti-psychotic prescribing | - resident agitation  - resident quality of life | 12 | 3 |
| Frade | Developing a learning set within dementia care: A practice development project | 2005 | Learning set/Reflective discussion of particular cases. 6 one-hour sessions. | + general positive feedback | + knowledge |  |  | 1 | 1 |
| Fruhauf, Jarrott & Lambert-Shute | Service-Learners at Dementia Care Programs: An Intervention for Improving Contact, Comfort, and Attitudes | 2004 | Classroom + practice project. Included dementia education and communication skills instruction, a structured activity (i.e., a scrapbook project to complete with the ADS clients), mid-semester meeting, and a student evaluation session at the end of the semester. |  | + confidence |  |  | 9 | 2 |
| Galvin, Kuntemeier, Al-Hammadi, Germino, Murphy-White & McGillick | “Dementia-friendly hospitals: care not crisis” an educational program designed to improve the care of the hospitalized patient with dementia | 2010 | Classroom based. Didactic lectures and group learning via review of case studies, generating care plans and discharge plans using forms specific to each institution. Modules included: Introduction, Medical overview, communication, dementia friendly care, and connecting the caregiver. | + largely useful and applicable | +/- knowledge but not maintained for some staff  + confidence  + attitudes |  |  | 7 | 2 |
| Goyder | Staff Training using STAR (Staff Training in Assisted Living Residences): A Pilot Study in UK Residential Care Homes (PART 2) | 2011 | Classroom + DVD based. Use of didactic teaching, discussion and group exercises with an emphasis on sharing experiences. Applied flexibly and interactively. The STAR DVD was an integral part, containing eight acted scenes with interactions between staff and residents, which depicted common behavioural problems. | + generally positive  + DVD felt to be particularly useful | + knowledge  +/- overall confidence not improved, sig change seen on ‘building relationships’ sub-scale only |  | + resident depression  + resident behaviours  - resident quality of life  - resident anxiety | 14 | 3 |
| Gozalo, Prakash, Qato, Sloane & Mor | Effect of the Bathing Without a Battle Training Intervention on Bathing-Associated Physical and Verbal Outcomes in Nursing Home Residents with Dementia: A Randomized Crossover Diffusion Study | 2014 | Train the trainer + DVD: Bathing without a battle. Trainers attended a 2-day joint training session, consisting of a detailed discussion of behavioural expressions of distress of nursing home residents during bathing, and approaches for preventing /managing distress. Trainers used the training CD ROM and DVD materials to train CNAs. |  |  | + type and duration of bath  + reduced anti-psychotic use | + resident agitation  + resident aggression | 13 | 3 |
| Hobday, Savich & Gaugler | An Internet-Based Multimedia Education Prototype to Enhance Late-Stage Dementia Care: Formative Research Results | 2010 | Online learning. Ten modules planned, 3 of which were fully written, reviewed and approved for this pilot study: introduction to dementia, rethinking activities and toileting. | + relevant to practice  - wanted more handouts, interactivity and opportunity to share learning with a group  - modules too slow | + knowledge  + confidence |  |  | 7 | 2 |
| Hobday, Savik, Smith & Gaugler | Feasibility of Internet Training for Care Staff of Residents with Dementia: The CARES® Program | 2010 | Online learning. Three initial sections combined into the opening module of foundational training: Opener, Introduction to dementia, Introduction to behavior management. Each of these sections delivered content via text, graphics, and video. | + most enjoyed learning experience  - content not dementia specific enough, too simple and did not include enough information  - struggles with technical access  - wanted a group training component | + knowledge  + confidence |  |  | 5 | 1 |
| Innes | Student-Centred learning and Person-centred dementia care. | 2001 | Classroom based. Person centred care and communication principles. Each participant completed two assessments based on work with a person of their choice. Encouraged staff to build upon good practices. Exercises to encourage staff to try out what they had discussed. | + liked group work  + session length OK  +/- improved over time as they became more interested | + knowledge  + confidence |  |  | 4 | 1 |
| Innes, MacKay & McCabe | Dementia studies online: reflections on the opportunities and drawbacks of eLearning | 2006 | Online learning. 10 modules, employed range of online teaching methods alongside face-to-face introductory sessions and telephone support. Web site included discussion boards, chat rooms, an online library with articles and videos, online help, and personal email. Module workbooks in hard copy supplements online learning resources. | + flexible, able to do at own pace  + discussion boards  - technical issues accessing materials  - learner isolation |  |  |  | 4 | 1 |
| Irvine, Ary & Bourgeois | An Interactive Multimedia Program to Train Professional Caregivers | 2003 | Individual DVD vs group DVD lecture - Interactive multimedia program or videotaped in-service lecture. Specific communication and behavior management skills presented (e.g., speaking, reacting, redirection, and use of communication cards). | + interactive multi-media (IMM) preferred to videotaped group lecture | + knowledge in IMM  + confidence in IMM |  |  | 11 | 3 |
| Irvine, Beaty, Seeley & Bourgeois | Use of a Dementia Training Designed for Nurse Aides to Train Other Staff | 2012 | Individual DVD, video based internet training program. Modules included Speaking Skills, Reacting Skills, Redirection, Communication Cards, and When Bad Things Happen. Included video-modeling vignettes, right-way and wrong-way exemplars, testimonials, and narration. | + highly rated by learners  + valued video clips used | + knowledge for nurses  + attitudes for nurses  + confidence non-nursing roles  - attitudes non-nursing roles |  |  | 11 | 3 |
| Isaacson, Safdieh & Ochner | Effectiveness of a modified Continuum curriculum for medical students: A randomized trial | 2011 | Standard clerkship training on dementia (slide presentation), and a condensed medical student version of the Continuum: Dementia training material developed by the American Academy of Neurology. | + positively rated | + knowledge |  |  | 11 | 3 |
| Jefferson, Cantwell, Byerly & Morhardt | Medical student education program in Alzheimer’s disease: The PAIRS Program | 2012 | Classroom + Buddy. 3 hours of lectures on Alzheimer's disease and monthly lunchtime lectures from guest lecturers on AD topics. Staff were paired with buddy with AD, and met them monthly for a minimum 4 hours of social or cultural visit for 6 months. | + like blend of lectures and experiential learning | + knowledge  + attitudes |  |  | 11 | 3 |
| Jeon, Luscombe, Chenoweth, Stein-Parbury, Brodaty, King & Haas | Staff outcomes from the Caring for Aged Dementia Care REsident Study (CADRES): A cluster randomised trial | 2012 | Classroom + sharing learning with colleagues. PCC training: used a train the trainer approach following Bradford University's PCC training manual. DCM training: used Bradford University's DCM training manual. Included 3 day PCC and DCM training, experts working alongside, and providing advice, guidance and telephone support. |  | - attitudes |  |  | 14 | 3 |
| Juola, Bjorkman, Pylkkanen, Finne-Soveri, Soini Kautiainen, Bell & Pitkala | Feasibility and baseline findings of an educational intervention in a randomized trial to optimize drug treatment among residents in assisted living facilities | 2014 | Classroom based. Education in geriatric pharmacotherapy. Consisted of lectures on harmful drugs, particularly in older people. Staff also received information about clinically significant (D-class) DDIs to be used in their day to day work. | + mostly positive |  |  |  | 4 | 1 |
| Kaf, Barboa, Fisher & Snavely | Effect of Interdisciplinary Service Learning Experience for Audiology and SpeechLanguage Pathology Students Working With Adults With Dementia | 2011 | In-service. In weekly visits, the speech-language pathology students participated in the residents’ activities of daily living to stimulate their verbal communication. Each class period included a class discussion of students’ experiences at the nursing home. |  | + attitudes |  |  | 9 | 2 |
| Kalsy, Heath, Adams & Oliver | Effects of training on controllability attributions of behavioural excesses and deficits shown by adults with Down syndrome and dementia | 2007 | Classroom based. The staff training programme was a 4-hour workshop comprising of experiential and didactic teaching methods, and workshop materials. |  | + knowledge |  |  | 7 | 2 |
| Kellett, Moyle, McAllister, King & Gallagher | Life stories and biography: a means of connecting family and staff to people with dementia | 2010 | Classroom + action learning. Participants completed the Family Biography Workshop, 6 weekly 2-hour sessions attended by staff, family member and the researcher. Workshops designed to help them build a biography of the person with dementia. |  | + knowledge  + attitudes | + person-centred care | + carer satisfaction  + resident outcomes (excluded from analysis) | 9 | 2 |
| Kelly | Changes in knowledge and attitudes of certified nursing assistants about ethics of treatment choices for nursing home residents with end-stage Alzheimer’s disease | 2004 | Classroom based. ‘Standing By You’, a 90-minute presentation using a didactic classroom-style method with lecture, overhead transparencies, and illustrative wall posters. Two additional 50-minute class support sessions to reinforce the didactic material about advance directives and decision-making. | + informal positive responses | + knowledge |  |  | 11 | 3 |
| Kemeny, Boettcher, DeShon & Stevens | Using Experiential Techniques for Staff Development: Liking, Learning, and Doing | 2006 | Classroom + in-service. Project RELATE: formal training intervention and informal coaching sessions delivered by a facilitator during a 5 week period. Based on the learning needs of staff and learning objectives. Experiential techniques such as role plays and simulations used for learning objectives involving interpersonal interaction. | + generally positive, liked experiential techniques | + knowledge |  |  | 9 | 2 |
| King, Kelder, Phillips, McInerney, Doherty, Walls, Robinson & Vickers | Something for Everyone: MOOC Design for Informing Dementia Education and Research | 2013 | MOOC – e-learning. Three primary themes: ‘the brain’, ‘the diseases’ and ‘the person’. Video clips of up to thirty minutes in combined duration; One or more reflective questions, to be entered into a ‘journal’; quiz about video content; questions to guide forum discussion; Other supporting materials. | + overall positive response |  |  |  | 0 | 1 |
| King, O’Brien, Edelman & Fazio | Evaluation of the Person-Centered Care Essentials Program: Importance of Trainers in Achieving Targeted Outcomes | 2011 | Classroom based. A two-day train-the-trainer conference, to prepare two representatives (“trainers”) from each participating institute to deliver the program to direct care workers (DCWs). The conference included training on delivering each of the four modules, PCC, adult learning principles, overcoming barriers, and implementing the evaluation. Trainers returned to their home institutes to train staff in 4x1 hour modules. |  | +/- knowledge in some modules but not others |  |  | 7 | 2 |
| Kontos & Naglie | Expressions of Personhood in Alzheimer’s Disease: An Evaluation of Research-Based Theatre as a Pedagogical Tool | 2007 | Live performance of a dramatic production which consisted of five separate vignettes that were thematically connected, in that all featured bodily expressions of selfhood by severely cognitively impaired residents of an Alzheimer support unit. |  | + knowledge |  |  | 7 | 2 |
| Kontos, Mitchell, Mistry & Ballon | Using Drama to Improve Person-Centred Dementia Care | 2010 | Classroom based + drama. A 12-week interprofessional arts-informed program to improve person-centred dementia care. Sessions were 2 hours per week. Utilised dialogue, critical reflection, role-play, and dramatized vignettes as educational modalities. Used DVD of a research-based production Expressions of Personhood in Alzheimer’s about embodied selfhood. | + valued vignettes | + knowledge |  |  | 13 | 3 |
| Kuske, Luck, Hanns, Matschinger, Angermeyer, Behrens & Riedel-Heller | Training in dementia care: a cluster-randomised controlled trial of a training programme for nursing home staff in Germany | 2009 | Classroom - Lectures, educational videos, PowerPoint, handouts, brainstorming, short games, scenarios, real life examples. Information about dementia, person and the environment, communication, self-reflection. |  | + knowledge | + reduced restraint use  - reduced anti-psychotic prescribing | - staff burnout | 12 | 3 |
| Lambert-Shute, Jarrott & Fruhauf | Service-Learning at Dementia Care Programs: An Orientation and Training Program | 2004 | Classroom + in-service. Students learned about causes of dementia, behavioral characteristics, and communication techniques. Included a structured scrapbook project for service-learners to exercise their new knowledge and skills. | + helpful and provided framework for working with people with dementia  + valued facilitators role play of strategies | + confidence |  |  | 9 | 2 |
| Landreville, Dicaire, Verrault & Levesque | A training program for managing agitation of residents in long-term care facilities description and preliminary findings | 2005 | Classroom + supervision. In-service includes five sessions, each lasting 90 minutes and focusing on a specific theme including communication and elements of agitation. Didactic approach included brief lectures, presentation slides, case examples, and exercises based on residents in the participants’ care units. Trainees use pre-determined questions to help them describe agitated behavior, and to design and evaluate an intervention. | + satisfied  + felt applicable to practice | + confidence |  |  | 6 | 2 |
| Lathren, Sloane, Hoyle, Zimmerman & Kaufe | Improving dementia diagnosis and management in primary care: a cohort study of the impact of a training and support program on physician competency, practice patterns, and community linkages | 2013 | Classroom based. Four one-day sessions, primarily discussion-based, and included case discussions. Examples of cognitive tool administration and scoring were provided. Participants received e-newsletters containing updates on dementia research and reminders about community resources. All participants received a binder of materials containing relevant information. |  | + confidence | + referrals |  | 7 | 2 |
| Lea, Marlow, Bramble, Andrews, Crisp, Eccleston, Mason & Robinson | Learning Opportunities in a Residential Aged Care Facility: The Role of Supported Placements for First-Year Nursing Students | 2014 | Classroom + in-service. Students participated in a 2-week placement. Separate weekly feedback and debriefing meetings facilitated by project staff were held with both mentors and students in addition to a 2- to 3-hour workshop that addressed issues around dementia and palliative care. |  | + knowledge  + confidence  + attitudes |  |  | 9 | 2 |
| Lea, Marlow, Bramble, Andrews, Eccleston, McInerney & Robinson | Improving student nurses’ aged care understandings through a supported placement | 2015 | Classroom based - Intervention used Wicking Teaching Aged Care Facilities Program (TACFP) to prepare mentors and students for placements. Mentors were trained in mentoring and dementia. Students attended a 2 hour training session on dementia. | + viewed as helpful | + knowledge |  |  | 7 | 2 |
| Lee, Weston & Hillier | Developing Memory Clinics in Primary Care: An Evidence-Based Interprofessional Program of Continuing Professional Development | 2013 | Classroom + mentorship. The training program for multidisciplinary FHT team members consists of a 2-day workshop aimed at increasing team knowledge and skill related to the assessment and management of cognitive impairment followed by a 3-day mentorship program. | + positive and applicable to practice | + knowledge  + confidence |  |  | 8 | 2 |
| Leone, Deudon, Bauchet, Laye, Bordone, Lee, Piano, Friedman, David, Delva, Brocker, Yesavage & Robert | Management of apathy in nursing homes using a teaching program for care staff: the STIM-EHPAD study | 2013 | Classroom + in-service with feedback. First intervention: 2-hr training on AD and behavioural and psychological symptoms of dementia (BPSD). Second: a weekly 4-h training for a month, on methods and practical advice on apathy and depression. Two hours on techniques for dealing with deficits in ADL. |  | +/- knowledge moderate but not significant improvement | - anti-psychotic prescribing | - resident behaviours  +/- resident Activities of daily living scores  +/- resident apathy | 12 | 3 |
| Lerner, Resnick, Galik & Russ | Advanced Nursing Assistant Education Program | 2010 | Classroom based. Lectures, case studies, learner participation. The daylong program consisted of six learning modules presented by three nurse faculties: Recognising acute medical problems in residents, Managing challenging behaviors, Dealing with difficult people, Restorative care philosophy, Infection control update, leadership. Participants were divided into three groups and worked to improve a problem within each of their facilities. | + created desire to undertake further learning | + knowledge |  |  | 6 | 2 |
| Lintern, Woods & Phair | Before and after training: a case study of intervention. | 2000 | Classroom based. Two day training course for senior staff, followed by a two day course for direct care staff. Training focused on developing person-centred dementia care. Uses a range of techniques including traditional lecturing, interactive discussions, role-play and group work. Some members of staff completed projects to develop a more individualised approach. |  | + attitude | + person-centred care | - resident well-being | 8 | 2 |
| Litvin, Davis, Moran, Iverson, Zhao & Zapka | The Use of Clinical Decision-Support Tools to Facilitate Geriatric Education | 2012 | Classroom + Decision support tool. Intensive 3-month education and quality improvement modules were devised for three selected ACOVE conditions. CDS tools were incorporated into the electronic medical record (EMR) to provide recommendations that residents could incorporate while caring for older adults. Also included didactic lectures and academic detailing by faculty. | + consistently reported as valuable |  | + use of assessment process |  | 9 | 2 |
| Liu, Pang & Lo | Development and implementation of an observational pain assessment protocol in a nursing home | 2012 | Classroom based. workshops on the C-PAINAD protocol. The workshops included (1) using and interpreting C-PAINAD, (2) recognising and responding to expressions of pain and (3) developing caregiving approaches to minimise pain during the nursing procedures. | + easy to use and follow  - not always easy to apply in practice | + confidence | - resident pain scores  - pain medication prescribing |  | 6 | 2 |
| Long | Pain Management Education in Long-Term Care: It Can Make a Difference | 2013 | Classroom based. Five education modules and onsite consultations on problem situations regarding pain. Staff learned about successful pain assessment and management from a variety of sources during the 6-month long program. |  | + knowledge  +/- attitudes only in qualified and not unqualified staff |  |  | 6 | 2 |
| MacDonald, Stodel & Casimiro | Online dementia care training for healthcare teams in continuing and long-term care facilities: a viable solution for improving quality of care and quality of life for residents | 2006 | Online + discussion: 8 week program offered through WebCT, a course management online system. Four modules: 2-4 pages of didactic prose followed by an online self-evaluation and individual and team exercises that were to be completed on the unit. | + materials clear, concise  - difficulties with computer skills, access to computers, technical usability of programme | + knowledge | + person-centred care |  | 9 | 2 |
| Mackenzie & Peragine | Measuring and enhancing self-efficacy among professional caregivers of individuals with dementia | 2003 | Classroom + simulation. Self-efficacy training developed using role-play scenarios. Consisted of four two hour modules on teamwork, challenging behaviour, family, and review. Each module consisted of one hour of didactic information and discussion, followed by one hour of experiential role-playing. | + positive, applicable | + knowledge |  | - staff emotional exhaustion  +/- staff accomplishment post but not maintained at follow-up | 9 | 2 |
| Magai, Cohen & Gomberg | Impact of Training Dementia Caregivers in Sensitivity to Nonverbal Emotion Signals | 2002 | Classroom + experiential. Nonverbal sensitivity training. The 10 units included the universal and culture-specific aspects of the basic emotions, personal emotional triggers, and training in emotion validation skills. | + enjoyed, felt more professional |  |  | - resident depression  - resident behaviours  - resident agitation  + resident positive mood | 11 | 3 |
| Mahendra, Freemant & Dionne | Teaching Future Providers about Dementia: The Impact of Service Learning | 2013 | Classroom + online. Aimed at building a strong knowledge base around dementia, specific approaches for assessment and management of PWD, and disparities in service provision to PWD in LTC settings. The novel concept of a “flipped classroom” was then utilised where students were provided detailed online content outside class, whereas in-class time was utilized for completing hands-on clinical assignments targeting assessment. | + liked hands on learning  - logistics of off-site learning | + knowledge  + attitude |  |  | 6 | 2 |
| Markert, O’Neill & Bhatia | Using a Quasi-experimental Research Design to Assess Knowledge in Continuing Medical Education Programs | 2003 | Programs followed the traditional didactic conference format of lectures and panels. 1-day conference. |  | + knowledge |  |  | 7 | 2 |
| McCabe, Bird, Davison, Mellor, MacPherson, Hallford & Seedy | An RCT to evaluate the utility of a clinical protocol for staff in the management of behavioral and psychological symptoms of dementia in residential aged-care settings | 2015 | Classroom + in-service vs in-service only. A two-hour training session in which staff were helped to work through and identify probably causal factors for the behavior of residents. Followed by a two-hour workshop providing an overview of dementia, BPSD, and person-centered care strategies. A mental health professional experienced in working with BPSD provided clinical support for training/ support and support conditions. |  | + confidence  + attitudes |  | - staff strain  + resident agitation decreased for training + in-service | 14 | 3 |
| McCaffrey, Tappen, Lichtstein & Friedland | Interprofessional education in community-based Alzheimer’s disease diagnosis and treatment | 2013 | Classroom + inservice. Information regarding the clinical manifestations, diagnosis and treatment of dementia and impact of the disease. Students were divided into medical student–nurse practitioner student dyads. Each dyad participated in five clinical experiences over a 15-week semester. | + valuable experience | + knowledge  + attitudes |  |  | 9 | 2 |
| McCarthy | An evaluation of the effectiveness of Dementia Care Essentials in improving the quality of residential and community aged care | 2012 | Classroom based. A program of three days, face-to-face training including workplace assessment and completion of tasks by participants in fulfilment of the criteria for unit competency (varies nationally). | + felt was high quality |  | +/- staff communication | - resident depression | 9 | 2 |
| McCurry, LaFazia, Pike, Logsdon & Teri | Development and Evaluation of a Sleep Education Program for Older Adults with Dementia Living in Adult Family Homes | 2012 | Classroom based: The SEP was designed to teach AFH caregiver-staff about non-pharmacological strategies to improve sleep in older adults with dementia, and to help them implement a realistic sleep plan for each resident. The SEP was delivered in 4 sessions over 4 weeks by an experienced trainer. | + interested and desire to apply | + knowledge | + assessment of resident behaviour | +/- resident sleep  - resident behaviours | 12 | 3 |
| McGilton, O’Brien-Pallas, Darlington, Evans, Wynn & Pringle | Effects of a Relationship-Enhancing Program of Care on Outcomes | 2003 | Classroom based. All supervisors (unit manager and charge nurses) took a 3-session educational program around providing support to care providers. Next was a five-session educational program to the care providers to teach skills necessary to provide effective relational care to residents. The sessions were 15-20 minutes. |  |  | + person-centred care | + more empathic care | 12 | 3 |
| McPhail, Traynor, Wikstrom, Brown & Quinn | Improving outcomes for dementia care in acute aged care: impact of an education programme | 2009 | Classroom based: 10 week education program of 1 hour sessions: What is Dementia; Delirium/ Depression/Dementia; Behaviour Management; Medications; Sedation Policy; Attitudinal Change; Sexual Dis-inhibition; Restraint; Poole’s Algorithm; Social Management in Acute Care. | + positive, applicable | + knowledge |  | + reduced aggressive behaviours recorded | 3 | 1 |
| Mitchell, Dupuis, Jonas-Simpson, Whyte, Carson & Gillis | The Experience of Engaging With Research-Based Drama: Evaluation and Explication of Synergy and Transformation | 2011 | Drama: a performance of I’m still here developed from five stories with persons living with dementia and one story with daughters whose mothers were diagnosed with dementia into an hour-long script. All studies were qualitative and focused on explicating persons’ lived experiences and quality of life. |  | + knowledge  + attitudes |  |  | 4 | 1 |
| Monette, Champoux, Monette, Fournier, Wolfson, du Fort, Sourial, Le Cruguel & Gore | Effect of an interdisciplinary educational program on antipsychotic prescribing among nursing home residents with dementia | 2008 | Classroom + written resource. Program included consciousness-raising, educational sessions, and clinical follow-up. The primary focus was on the need for non-pharmacological approaches to treatment of disruptive behaviors. Summary pamphlets and educational guides. Clinical follow up by pharmacists. |  |  | + anti-psychotic discontinuation/dose reduction  - prescribing other psychotropics  - restraint use | - staff stressful events  + resident behaviours | 12 | 3 |
| Monette, J., Monette, M., Sourial, N., Vandal, A. C., Wolfson, C., Champoux, N., Fletcher, J., & Savoie, M. L. | Effect of an Interdisciplinary Educational Program on Antipsychotic Prescribing Among Residents With Dementia in Two Long-Term Care Centers | 2013 | Classroom based. See above. |  |  | +/- anti-psychotic prescribing, decreased after training but then increased in one centre and continued to decrease in other  - no change other psychotropic prescribing | - resident behaviours  - staff stress | 13 | 3 |
| Moorhouse & Hamilton | Not If, But When: Impact of a Driving and Dementia Awareness and Education Campaign for Primary Care Physicians | 2014 | Written resource. aims to guide physicians through the process of driving cessation in dementia. Informative materials plus printable information sheets and checklists for caregivers. |  | - confidence | + assessment and discussion of driving capacity |  | 10 | 2 |
| Narevic, Giles, Rajadhyax, Managuelod, Monis & Diamond | The effects of enhanced program review and staff training on the management of aggression among clients in a long-term neurobehavioral rehabilitation program | 2011 | Learning set + case scenarios. Introduced ongoing post-incident review and training and the provision of CNA support groups. Groups’ goals are to increase the awareness of factors contributing to clients’ behaviors; increase staff self-efficacy; and facilitate CNAs sharing their knowledge with each other and management staff. |  |  | + weekly review meetings held | + reduction in aggressive incidents | 6 | 2 |
| National Museums Liverpool | An evaluation of National Museums Liverpool: Dementia Training Programme | 2012 | Classroom: House of Memories is a training and delivery programme built around the objects, archives and stories held within the Museum of Liverpool. It aims to provide social and health care staff with new skills and resources to share with people living with dementia. | + high satisfaction with training | + knowledge  + attitudes |  |  | 11 | 3 |
| Nayton, Fielding, Brooks, Graham & Beattie | Development of an Education Program to Improve Care of Patients With Dementia in an Acute Care Setting | 2014 | Classroom based. Seven sessions of 25 min, included: Neurobiology and Person-Centered Care, Communication Strategies and the Acute Care Environment. The facilitator framed discussions and content messages within the theme of the ‘View From Here’ (i.e., from the viewpoint of the person with dementia). | + high satisfaction with training, relevant  - wanted longer sessions | + knowledge  + confidence |  |  | 6 | 2 |
| O'Shea, Devane, Cooney, Casey, Jordan, Hunter, Murphy, Newell, Connolly & Murphy | The impact of reminiscence on the quality of life of residents with dementia in long-stay care | 2014 | Classroom + mentorship. Staff trained to incorporate reminiscence strategies when developing care plans for residents with dementia. A structured education programme, facilitated by experienced nurse educators, delivered over 3 days with telephone support and one site visit. |  |  |  | + resident quality of life (per protocol but not intention to treat analysis) | 9 | 2 |
| O'Sullivan & Hocking | Translating knowledge into practice: An exploratory study of dementia-specific training for community-based service providers | 2015 | Classroom based. Two training workshops of 3.5 to 4 hours duration, one week apart. A combination of quizzes, reflective exercises, and group discussion. The exercises were supported by stories grounded in practice. Used a training manual based on national policy documents and strategies as well as resources, ideas, and topics that underpin international approaches to training. | + relevant materials  + manual provided was deemed helpful resource |  | + person-centred care |  | 11 | 3 |
| Palmer, Lach, McGillick, Murphy-White, Carroll, & Armstrong | The Dementia Friendly Hospital Initiative Education Program for Acute Care Nurses and Staff | 2014 | Classroom based. 5 modules of didactic content, slides, videos, learning activities, and handouts. Standard slides covering key topics used consistently. The slides included video clips of professionals addressing problems associated with dementia. A binder with copies of the slides and handouts of important information given to participants after training. | + rated as effective, information covered was useful and helpful to role  + liked handouts provided and use of video clips to demonstrate concepts  + trainer knowledge | + knowledge  + confidence  + attitude |  |  | 8 | 2 |
| Parks, Haines, Foreman, McKinstry & Maxwell | Evaluation of an Educational Program for Long-term Care Nursing Assistants | 2005 | Classroom based. Five educational seminars: (1) introduction to the program and dementia as a terminal disease, (2) issues in pain control, (3) issues pertaining to non-pain symptoms at the end of life, (4) issues regarding feeding, and (5) issues regarding psychology and spirituality. Includes a take-home self-study module that includes review questions. |  | + knowledge  + attitude |  |  | 9 | 2 |
| Passalacqua & Harwood | VIPS Communication Skills Training for Paraprofessional Dementia Caregivers: An Intervention to Increase Person-Centered Dementia Care | 2012 | Classroom, workshops. The intervention was offered in four 1-hour workshops over a period of 4 weeks. Each session featured an explanation of the week’s concept (V, I, P, or S) and evidence-based communication skills training. Workshops included videotaped vignettes, power point slides, discussion, group and dyadic activities, role-playing and guided visualization exercises. | + largely positive, lots of information, fun and interactive  + tips and techniques useful  - not like homework  - some not like role-playing | + attitude | + staff communication more person-centred |  | 9 | 2 |
| Pellfolk, Gustafson, Bucht & Karlsson | Effects of a Restraint Minimization Program on Staff Knowledge, Attitudes, and Practice: A Cluster Randomized Trial | 2010 | Group DVD. Monthly themes: Dementia, Delirium in old People; Falls and fall prevention; Use of physical restraints; Caring for people with dementia; Complications in dementia. One volunteer from each unit attended the whole education program compressed into 2 days of seminars. The remaining staff received their education in six 30-minute videotaped lectures. Three of the lectures also included a clinical vignette for group discussions. |  | +/- knowledge in some topics not others  - attitude | + restraint use  - anti-psychotic prescribing | - resident falls | 11 | 3 |
| Pereles, Lockyer, Ryan, Davis, Spivak & Robinson. | The use of the opinion leader in continuing medical education | 2003 | Classroom + practice project. Lecture and group discussions on the current concepts about being an opinion leader, adult education principles, and review of the educational content in a two-hour course. Team building exercises to help OLs get to know each other. Community projects extended over one year. Two-hour JHF/AGS course, "Memory loss: Evaluation in primary care". | - role a challenge and ambiguous  - felt not enough dementia expertise provided | + knowledge | + assessment |  | 7 | 2 |
| Peterson, Berg-Weger, McGillick & Schwartz. | Basic Care I: The effect of dementia-specific training on certified nursing assistants and other staff | 2002 | Classroom based. Lecture, video, role play, sensitisation exercises, and interactive discussion. Six-hour class of practical techniques to help with activities of daily living and understanding of the physiology of dementia, common behaviors exhibited by people with dementia. |  | + knowledge  - confidence |  |  | 11 | 3 |
| Resnick, Cayo, Galik & Pretzer-Aboff | Implementation of the 6-Week Educational Component in the Res-Care Intervention: Process and Outcomes | 2009 | Classroom vs classroom + in-service. The 6 week Res-Care Intervention for nursing assistants around restorative care. 30 minute weekly education sessions. Group discussion and interaction. Facilitators working through examples of poor care in practice. Self efficacy techniques. Flowsheets and worksheets. | + interactivity  + use of case studies  + opportunity to demonstrate and practice skills | + knowledge |  |  | 10 | 2 |
| Richardson, Kitchen & Livingstone | What staff know about elder abuse in dementia and the effect of training | 2004 | Classroom + written materials for identification and management of all types of abuse. Use of vignettes. Group 1 attended an educational course commissioned by the employing National Health Service (NHS) trust and local Social Services department. Group 2 were given reading material with the same content as the course. |  | + knowledge in face-to-face training  - knowledge in learning by reading |  |  | 7 | 2 |
| Richardson, Kitchen, & Livingston | The effect of education on knowledge and management of elder abuse: a randomized controlled trial | 2002 | Classroom + written materials. See above for description. |  | + knowledge in face-to-face training  - knowledge in learning by reading |  |  | 9 | 2 |
| Roberts & Gaspard | A palliative approach to care of residents with dementia | 2013 | Classroom based. The CHPCA (2002) dimensions of palliative care formed the core components of the workshop. Reflection on assumptions about people with dementia and palliative care, considering alternative care strategies. Participants recalled practice and personal experiences; video clips; small group discussion; use of props; writing commitment to change statements. |  | + knowledge  + confidence |  |  | 6 | 2 |
| Rodriguez, Marquett, Hinton, McBride, & Gallagher-Thompson | The Impact of Education on Care Practices: An Exploratory Study of Whether “Action Plans” Influence Health Professionals' Behavior | 2010 | Classroom + practice project. Seven hour lecture-based conference with Question and Answer segments and Resource Fairs. In the second study, the workshop included 8-hours of intensive discussion, role-playing, and problem-solving. Action planning. |  |  | + person-centred care |  | 7 | 2 |
| Ruiz, Smith, van Zuilen, Williams, & Mintzer | The Educational Impact of a Computer-Based Training Tutorial on Dementia in Long Term Care for Licensed Practice Nursing Students | 2006 | Individual CD-ROM, composed of 7 30-minute training modules: 1. Understanding Dementia 2. Communication 3. Distress Behaviors 4. Loved Ones 5. Activities of Daily Living 6. Environment 7. Ethics. Incorporates a variety of presentation formats including text, animations, video, audio, and interactive exercises. | + positive, easy to use | + knowledge  + confidence |  |  | 12 | 3 |
| Sackley, Rodriguez, Berg, Badger, Wright, Besemer, Reeuwijk & Wely | A phase II exploratory cluster randomized controlled trial of a group mobility training and staff education intervention to promote urinary continence in UK care homes | 2008 | Classroom based. Staff education component comprised 2-hour workshops on continence care and mobility care. The mobility training protocol for residents was targeted at maintaining and improving mobility and incorporated continence promotion – a group exercise class over 4 weeks. | + good attendance, positive learner views  + felt need for further training |  |  | + resident continence  + resident mobility | 10 | 2 |
| Schindel-Martin, Morden, Cetinski, Lasky, McDowell & Roberts | Teaching staff to respond effectively to cognitively impaired residents who display self-protective behaviors. | 2003 | Classroom based. A 7½ hour workshop for small staff groups. Included a component of the NCI curriculum, specifically appropriate release techniques and containment holds. Short lecture format, experiential exercises, dementia-specific videotapes, demonstration of appropriate techniques, and supervised role play. |  | + knowledge |  |  | 9 | 2 |
| Schlaudecker, Lewis, Moore, Pallerla, Stecher, Wiebracht & Warshaw | Teaching Resident Physicians Chronic Disease Management: Simulating a 10- Year Longitudinal Clinical Experience With a Standardized Dementia Patient and Caregiver | 2013 | Classroom based. Over 3 half-day sessions, an unfolding SP case was implemented to simulate a 10-year longitudinal relationship between physicians and a person with dementia. Use of lectures, videotaped longitudinal SP stations, SP feedback sessions, case discussion, and video review. | + rated positively, valuable  + scenario was realistic | + knowledge  + confidence | + staff person-centred communication |  | 8 | 2 |
| Schrijnemaekers, van Rossum, Candel, Frederiks, Derix,Sielhorst & van den Brandt | Effects of emotion-oriented care on elderly people with cognitive impairment and behavioral problems | 2002 | Classroom based, six days (with intervals). Lectures on dementia and care models for communicating with people with dementia (e.g. Validation, Reminiscence, sensory stimulation), implementation of practical skills. Methods such as teaching, homework, class assignments and exercises, role-playing and video-presentations. | + positive reaction, good attendance |  |  | - resident behaviours  - resident activities of daily living  - resident communication | 13 | 3 |
| Sidani, LeClerc & Streiner | Implementation of the abilities-focused approach to morning care of people with dementia by nursing staff | 2009 | Classroom based. 2-hour session around the abilities-focused approach to morning care. Topics covered using a mix of didactic presentation supported by audio-visual materials and group discussion of the content, illustrated with case examples. Written materials supplied. |  |  | + person-centred care |  | 12 | 3 |
| Sidani, Streiner, & LeClerc | Evaluating the effectiveness of the abilities-focused approach to morning care of people with dementia | 2012 | Classroom based. Focused on instructing nurses in the effects of dementia on social and self-care activities: the AFMC components; the principles for selecting appropriate AFMC interventions; and the advantages of the abilities-focused approach to care to residents and nurses. A list of the strategies and support from advanced practice nurses (APNs). |  |  | + person-centred care | - resident agitation  - resident activities of daily living  - resident physical and psychosocial functioning | 10 | 2 |
| Skaalvik, Normann & Henriksen | Student experiences in learning person-centred care of patients with Alzheimer’s disease as perceived by nursing students and supervising nurses | 2010 | Classroom + in-service. SNs were given 10 weeks clinical practice in care for the elderly in a TNH. Students received lectures on person-centred care. The lectures focused on validation and RT. The SNs had the opportunity to participate in domestic teaching (DT) on person-centred care of patients with AD. |  | - knowledge |  |  | 12 | 3 |
| Skog, Negussie & Grafström | Learning dementia care in three contexts: practical training in day-care, group dwelling and nursing home | 2000 | In-service. 18 licensed practical nurses (LPNs) received specialized training to become caregivers and mentors in the field of dementia care. |  | +/- knowledge gains were dependent on service setting |  |  | 9 | 2 |
| Sloane | Effect of a Person-Centered Mouth Care Intervention on Care Processes and Outcomes in Three Nursing Homes | 2013 | Classroom + in-service. Initial training consisted of person-centred seminars on oral pathology, dementia care, and individualized care planning plus skills training. The trainers provided care alongside the CNAs, using a peer-to-peer approach in which they solved problems as a team. Training and supervision daily for 2 weeks which reduced gradually. |  |  | + staff tooth brushing performance | + resident oral cleanliness  - resident food intake  - resident mouth/gum inflammation | 9 | 2 |
| Smythe, Jenkins, Harries, Atkins, Miller, Wright, Wheeler, Dee, Bentham & Oyebode | Evaluation of dementia training for staff in acute hospital settings | 2014 | Classroom + In-service. Written manual to ensure implementation fidelity. Each session involved working alongside the staff member and subsequent feedback and reflection over a six-week, classroom-based rolling programme. Content included delirium, managing behaviour perceived as challenging, nutrition and hydration, the importance of activities, falls management and end of life care. |  | - knowledge  - confidence  - attitudes |  |  | 9 | 2 |
| Söderlund, Norberg & Hansebo | Validation method training: nurses’ experiences and ratings of work climate | 2014 | Classroom + in-service + supervision. 10 days of theoretical VM training with monthly supervision, as well as practical VM training included in everyday work. Nurses documented practice via videotape and written reflections, with individual feedback from the supervisor. The programme ended with a written test about the VM, which led to VM certification. | + stimulating  + use of video particularly helpful  - too demanding, would not have taken part if known at outset what involved | + confidence |  |  | 13 | 3 |
| Speziale, Black, Coatsworth-Puspoky, Ross, & O’Regan. | Moving Forward: Evaluating a Curriculum for Managing Responsive Behaviors in a Geriatric Psychiatry Inpatient Population | 2009 | Classroom based. 7.5-hr GPA training course, multiple sessions over a 3-month period. Four modules: principles of person-centered care; the impact of dementia on the brain; strategies for responding to escalating behaviors; body containment principles. | + positively rated and would recommend to co-worker | + knowledge | + restraint use | + resident aggressive behaviours | 7 | 2 |
| Stevens-Roseman & Leung | Enhancing Attitudes, Knowledge, and Skills of Paraprofessional Service Providers in Elder Care Settings | 2004 | Classroom + simulation. Six training modules: relationship-building with older adults, communication, the effects of chronic pain, recognizing depression, working with older clients and family, and self-care. Written resource module entitled “community resources for older adults who live alone.” Sessions were experiential as well as didactic, drawing upon trainees’ experiences and challenges. | + beneficial | - knowledge  - attitude |  |  | 10 | 2 |
| Struck, Bernard & Teasdale | Effect of a Mandatory Geriatric Medicine Clerkship on Third-Year Students | 2005 | Classroom + in-service. 4-week clerkship. Students also participated in three weekly structured didactic sessions. The sessions covered the Geriatrics Syllabus Review, participation in the departmental journal club, and problem-based learning with a faculty facilitator. | + Problem-based learning  - didactic lectures | + knowledge  + confidence |  |  | 8 | 2 |
| Surr, Smith, Crossland & Robins | Impact of a person-centred dementia care training programme on hospital staff attitudes, role efficacy and perceptions of caring for people with dementia: A repeated measures study | 2015 | Classroom based. Cascade model. Two levels of training (Foundation and Intermediate), delivered over a total of 3.5 days. A series of seven 30-min modules covering person-centred care topics. Staff also received a train-the-trainer day to deliver sessions from the Foundation level PCTAH to peers for feedback. |  | + attitudes  + confidence |  |  | 13 | 3 |
| Szymczynska & Innes | Evaluation of a dementia training workshop for health and social care staff in rural Scotland | 2011 | Classroom based, workshops. Included speakers presenting on service improvement, provision of services, and rural dementia research. Group exercises included a case study analysis, a problem solving task, and a discussion of what it might mean to live with dementia. | + high satisfaction | + knowledge |  |  | 3 | 1 |
| Tannazzo, Breuer, Williams & Andreoli | A Dementia Training Program to Benefit Certified Nurse Assistant Satisfaction and Nursing Home Resident Outcomes | 2008 | Classroom based. 6-modules: 1. Putting the person first in dementia care. 2. The environment. 3. Enhancing the bathing experience. 4. Assisting a person with dementia with activities of daily living. 5. Mealtimes and the person with dementia. 6. Meeting the challenges of catastrophic reactions. Each conducted during a 3-hour period. 3 days of training per CNA over 3 weeks. |  | + knowledge |  | - staff overall job satisfaction  + staff intrinsic job satisfaction  - resident health complaints and insomnia  + resident facial expressions and physical movement  + resident withdrawal  + resident behaviours | 9 | 2 |
| Teresi, Ramirez, Silver, Boratgis, Kong, Eimicke, Pillemer & Lachs | A staff intervention targeting resident-to-resident elder mistreatment (R-REM) in long-term care increased staff knowledge, recognition and reporting: Results from a cluster randomized trial | 2013 | Classroom based. Three distinct sessions: (1) recognition and risk factors, (2) management, and (3) implementation of guidelines. Teaching contextualized information in the day-to-day work environment of the participants. |  | + knowledge | + staff conduct of behavioural assessments |  | 13 | 3 |
| Teri, Huda & Gibbons | Improving Dementia Care in Assisted Living Residences: Addressing Staff Reactions to Training | 2009 | Classroom + in-service. Training on-site at each ALR: 2x 4-hour group workshops using multiple methods to stimulate learning, including didactic content, case studies, discussion, and group exercises; and 4x 1-hour individualized sessions that allowed on-the-job practice of training skills. 3 brief sessions to help staff identify factors within the environment and their own actions to alter to enhance care. | - don’t have time to implement  - not part of job role  - conflicts with prior knowledge/training  + extensive discussion of facilitator approaches to addressing barriers |  |  |  | 8 | 2 |
| Teri, McCurry, Logsdon & Gibbons | Training Community Consultants to Help Family Members Improve Dementia Care: A Randomized Controlled Trial | 2005 | Classroom + in-service. Consultants trained via 2-hour orientation and manual. Additional reading and videotape materials describing the Seattle Protocols and general strategies for managing dementia-related behaviour. Consultants met with caregivers in their homes for 8 weekly sessions and four monthly phone calls. |  | + knowledge |  | + resident quality of life  + resident behaviours  + staff depression  + staff burden | 8 | 2 |
| Teri, Huda, Gibbons, Young & van Leynseele | STAR: A Dementia-Specific Training Program for Staff in Assisted Living Residences | 2005 | Training: see Teri et al., 2009 above. Content was systematic and standardized, yet flexible to the specific needs of staff, residents, and the particular ALR. Included lecture and discussion, roleplaying, observation of video case vignettes, overheads, and handouts. Group sessions to share experiences & individualized sessions to reinforce workshop materials. |  |  |  | - staff job satisfaction  + resident behaviours  + resident depression  + resident anxiety | 10 | 2 |
| Testad, Aasland & Aasland | The effect of staff training on the use of restraint in dementia: a single-blind randomised controlled trial | 2005 | Classroom + in-service. Six-hour seminar focusing on dementia, aggression, problem behaviour, decision making process and alternatives towards use of restraint. A manual was developed to standardise content. Each group received guidance for one hour every month, for six months. |  |  | + restraint use |  | 7 | 2 |
| Testad, Mekki, Førland, Øye, Tveit Jacobsen & Kirkevold | Modelling and evaluating evidence-based continuing education program in nursing home dementia care (MEDCED)—training of care home staff to reduce use of restraint in care home residents with dementia. A cluster randomized controlled trial | 2015 | Classroom based. 7 step guidance group: staff chose a situation including use of restraint and the DMP model (Testad, 2004) to emphasize relationships between resident and care staff and support the effective management of unmet need to reduce use of restraint and improve care. Training delivery over 7 months for intervention group. 2 day seminar (16 hours). 1 hour monthly 7 step guidance groups over 6 months. |  |  | - restraint use  - antipsychotic use | - resident agitation | 10 | 2 |
| Thompson & Devenney | Training in dementia for primary care professionals: the role ofthe Admiral Nurse | 2007 | Classroom based. Training included a combination of formal presentations, discussion and interactive workshops. Programme covered a wide range of topics. In phase 2, Family carers were involved in the workshops for administrative staff and some clinicians. Handouts kept to a minimum but presented in a folder for reference. | + useful, high quality  + carer input into training viewed positively |  | + quality and number of referrals to Admiral Nurse teams |  | 3 | 1 |
| van der Kooij, Dröes, de Lange, Ettema, Cools & van Tilburg. | The implementation of integrated emotion-oriented care: Did it actually change the attitude, skills and time spent of trained caregivers? | 2013 | Classroom + in-service. A large group of caregivers and staff members learned to apply IEOC through didactic and on-the-job training; one in four received a follow-up to learn to apply advanced IEOC; for every 15 residents, one nursing caregiver was trained to be a coach-consultant. Elements of psychosocial methods, such as Validation, Snoezelen and Reminiscence taught. |  | + knowledge | + person-centred care |  | 12 | 3 |
| van Zuilen, Mintzer, Milanez, Kaiser, Rodriguez, Paniagua, Ruiz & Roos | A Competency-Based Medical Student Curriculum Targeting Key Geriatric Syndromes | 2008 | Classroom based. The instructional activities and related competency assessments were introduced at various time points throughout the four-year curriculum. Faculty developed 25 learning objectives for dementia, 22 for falls, and 14 for delirium based on the FCGME competencies. | + high satisfaction | + attitude (excluded from analysis) |  |  | 6 | 2 |
| Vanlaere, Timmermann, Stevens & Gastmans | An explorative study of experiences of healthcare providers posing as simulated care receivers in a ‘care-ethical’ lab | 2012 | Simulation. Empathy sessions, aimed at care providers’ empathic skills. A two-day and one-night session in which eight care providers receive simulated care as patients. They receive care from undergraduate nurses (bathing, feeding, caring, recreation, etc.). Time dedicated to discussing and reflecting on what the care providers and undergraduates experienced during the empathy session. |  | + knowledge  + attitudes | + person-centred care |  | 12 | 3 |
| Velzke | Evaluation of a dementia care learning programme | 2014 | Classroom based, train-the-trainer. Trains and supports facilitators to train staff in dementia care over six-months. Staff who complete the course produce a reflective exercise from their own work experiences. Integrates the Promoting Excellence Framework (Scottish Government) and Standards of Care for Dementia in Scotland, and references dementia strategies and the common core principles. | + positively views by participants  - some examples provided ambiguous  - course too long  - needs adapting for use in daycare | + knowledge | + person-centred care |  | 8 | 2 |
| Vida, Monette, Wilchesky, Monette, Friedman, Nguyen, Dastoor, Cristache, Sourial, Tremblay & Gore | A long-term care center interdisciplinary education program for antipsychotic use in dementia: program update five years later | 2012 | Classroom based. two lectures, approx. 60–90 minutes, on pharmacological and nonpharmacological approaches to BPSD, and a four-step approach to managing these behaviours. |  |  | + antipsychotic prescribing |  | 10 | 2 |
| Vollmar, Mayer, Ostermann, Butzlaff, Sandars, Wilm & Rieger | Knowledge transfer for the management of dementia: a cluster-randomised trial of blended learning in general practice | 2010 | On-line + discussion vs classroom lecture + discussion. Presentation of the guideline content with regard to diagnosis, management, and therapy of dementia either by blended learning or by face-to-face teaching. A structured case discussion featured in face-to-face teaching in the QC meeting. | + blended on-line learning with discussion groups preferred to lecture and discussion  - only 56% of learners completed on-lien training | + knowledge (both learning methods) |  |  | 11 | 3 |
| Warshaw, Modawal, Kues, Moore, Margolin, Sehgal, Mueller & Cluxton | Community Physician Education in Geriatrics: Applying the Assessing Care of Vulnerable Elders Model with a Multisite Primary Care Group | 2010 | Distance + online. Office-based education, distribution of chart and patient education materials, physician follow-up mailings and case ‘‘quizzes,’’ and office-based quality improvement (QI) projects. Online modules covered medication management, falls and mobility, urinary incontinence, and dementia. Audience response system (ARS) used for learner engagement & discussion. | + general positive perceptions of training | + knowledge |  |  | 3 | 1 |
| Wesson & Chapman | A dementia education scheme | 2010 | A two-hour session covering: An overview of dementia; local and national dementia guidelines, tools, assessments, management and medication, acute confusion; pain assessment, monitoring and management; palliative care; life story books; general communication tips; Mental Capacity Act; carers’ support. | + staff appreciated the training  + structured charts and assessment particularly useful |  | + use of assessment charts  + antipsychotic prescribing |  | 1 | 1 |
| Westmoreland, Counsell, Tu, Wu & Litzelman | Web-Based Training in Geriatrics for Medical Residents: A Randomized Controlled Trial Using Standardized Patients to Assess Outcomes | 2010 | Web-based instruction vs written resource with same content. Modules covered four topics: dementia, depression, falls, and urinary incontinence. Case-based instruction, with a case unfolding through the eight sections. Modules were textual, with pictorial content. Video streaming included to demonstrate use of assessment tools. |  | + knowledge via web-based learning  - knowledge via paper-based learning | - staff conduct of assessments |  | 12 | 3 |
| Wilcock, Iliffe, Griffin, Jain, Thuné-Boyle, Lefford & Rapp | Tailored educational intervention for primary care to improve the management of dementia: the EVIDEM-ED cluster randomized controlled trial | 2013 | Classroom, practice-based workshops designed to elicit and assess the educational needs for the team as a whole. Initial needs assessment group discussion, from which an educational prescription was generated and supported with workshops and electronic information resources. |  |  | - staff assessment and recording of dementia  - dementia management approaches |  | 10 | 2 |
| Williams | Improving Outcomes of Nursing Home Interactions | 2006 | Classroom based. Three 1-hour educational sessions within a 2-week period in each facility, using an established program (Williams, Kemper, & Hummert, 2004). Effective versus ineffective strategies in communication, taking a resident perspective on communication, and identification of personal communication styles. |  |  | +/- staff communication approaches – some techniques improved others did not |  | 13 | 3 |
| Zimmerman, Mitchell, Reed, Preisser, Fletcher, Beeber, Reed, Gould, Hughes, McConnell, Corazzini, Lekan & Sloane | Outcomes of a Dementia Care Training Program for Staff in Nursing Homes and Residential Care/Assisted Living Settings | 2010 | Classroom based. Highly standardised, scripted training. Each session 45-55 min average. The evaluation focused on 3 modules in 6 sessions: Learning to lead; About dementia (one session on communication challenges in dementia and strategies to improve communication between residents and staff); Reducing pain. |  | +/- knowledge – increased for some topics but not others  - attitude | - staff communication  - person-centred care | - increased staff work stress | 12 | 3 |
| Zwijsen, Gerritsen, Eefsting, Smalbrugge, Hertogh & Pot | Coming to grips with challenging behaviour: A cluster randomised controlled trial on the effects of a new care programme for challenging behaviour on burnout, job satisfaction and job demands of care staff on dementia special care units | 2015 | Classroom based. GRIP: the use of structured forms explained and care staff educated on how to detect and reflect on signs of challenging behaviour. GRIP consists of four steps; detection, analysis, treatment and evaluation. |  |  |  | + staff job satisfaction  - staff exhaustion  - staff job demands | 13 | 3 |
